# Phosphatidylserine Targets Single-Walled Carbon Nanotubes to Professional Phagocytes *In Vitro* and *In Vivo*

**DOI:** 10.1371/journal.pone.0004398

**Published:** 2009-02-09

**Authors:** Nagarjun V. Konduru, Yulia Y. Tyurina, Weihong Feng, Liana V. Basova, Natalia A. Belikova, Hülya Bayir, Katherine Clark, Marc Rubin, Donna Stolz, Helen Vallhov, Annika Scheynius, Erika Witasp, Bengt Fadeel, Padmakar D. Kichambare, Alexander Star, Elena R. Kisin, Ashley R. Murray, Anna A. Shvedova, Valerian E. Kagan

**Affiliations:** 1 Center for Free Radical and Antioxidant Health, Graduate School of Public Health, Department of Environmental and Occupational Health, University of Pittsburgh, Pittsburgh, Pennsylvania, United States of America; 2 Department of Cell Biology & Physiology, University of Pittsburgh, Pittsburgh, Pennsylvania, United States of America; 3 Department of Chemistry, University of Pittsburgh, Pittsburgh, Pennsylvania, United States of America; 4 Pathology/Physiology Research Branch, Health Effects Laboratory Division (HELD), National Institute for Occupational Safety and Health (NIOSH), Morgantown, West Virginia, United States of America; 5 Clinical Allergy Research Unit, Department of Medicine Solna, Karolinska Institutet, Stockholm, Sweden; 6 Division of Biochemical Toxicology, Institute of Environmental Medicine, Karolinska Institutet, Stockholm, Sweden; University of Helsinki, Finland

## Abstract

Broad applications of single-walled carbon nanotubes (SWCNT) dictate the necessity to better understand their health effects. Poor recognition of non-functionalized SWCNT by phagocytes is prohibitive towards controlling their biological action. We report that SWCNT coating with a phospholipid “eat-me” signal, phosphatidylserine (PS), makes them recognizable *in vitro* by different phagocytic cells - murine RAW264.7 macrophages, primary monocyte-derived human macrophages, dendritic cells, and rat brain microglia. Macrophage uptake of PS-coated nanotubes was suppressed by the PS-binding protein, Annexin V, and endocytosis inhibitors, and changed the pattern of pro- and anti-inflammatory cytokine secretion. Loading of PS-coated SWCNT with pro-apoptotic cargo (cytochrome c) allowed for the targeted killing of RAW264.7 macrophages. *In vivo* aspiration of PS-coated SWCNT stimulated their uptake by lung alveolar macrophages in mice. Thus, PS-coating can be utilized for targeted delivery of SWCNT with specified cargoes into professional phagocytes, hence for therapeutic regulation of specific populations of immune-competent cells.

## Introduction

One of the major biomedical applications of carbon nanotubes (CNT) is their use as nanovectors in drug delivery paradigms. Professional phagocytes, particularly macrophages, are very attractive targets for selective drug delivery because these cells: i) host a variety of pathogens with significant public health impact, ii) play a critical role as orchestrators of inflammation as they regulate the production and release of pro- and anti-inflammatory mediators, reactive oxygen (ROS) and nitrogen species (RNS), particularly after exposure to particles [1], and iii) are significant contributors to the distribution of CNT in the body thus determining their potential toxic effects [2], [3]. Importantly, non-functionalized nanotubes are poorly recognized by macrophages *in vitro* and *in vivo* resulting in the avoidance of CNTs from macrophages-mediated “surveillance” [4], [5]. In contrast, functionalization of nanotubes induces their recognition by professional and non-professional macrophages and other cells [6]–[9]. However, the universal nature of the engulfment of covalently functionalized CNT by different types of cells precludes the possibility of their targeted delivery to specific cells [9]. This stimulated new lines of research on targeted interfacing of single walled carbon nanotubes (SWCNT) with living cells through specific coatings mimicking the cell surface [10]. In particular, glycopolymers - that mimic cell surface mucin glycoproteins and facilitate carbohydrate receptor interactions - have been developed to stimulate targeted engulfment of SWCNT by specific types of cells [10], [11].

Macrophage recognition and uptake of apoptotic cells (also termed “efferocytosis”) is an important type of cell/cell communications regulating inflammation [12], [13]. This interaction triggers not only effective clearance of apoptotic cells but also suppression of the inflammatory response [14], [15] or of adaptive immunity [16], thus limiting local tissue responses and normally leading to a quiet cell removal [13]. In contrast, inefficient apoptotic cell clearance is pro-inflammatory and pro-immunogenic. The recognition of apoptotic cells by macrophages is largely dependent on the appearance on the cell surface of an anionic phospholipid, phosphatidylserine (PS), which is normally confined to the cytosolic leaflet of plasma membrane [17], [18]. Thus, externalization of PS during apoptosis generates an “eat-me” signal for macrophages. Notably, non-apoptotic cells with externalized PS can also be taken up by macrophages and suppress ROS and RNS production [19]. PS externalization is one of several features contributing to recognition of apoptotic cells. Chemotactic factors such as lyso-PC [20], and bridging molecules such as MFG-E8 [21] are some examples of other important participants in the process of apoptotic cell clearance. Moreover, additional recognition signals on the surface of apoptotic cells have also been shown to be involved in this process, including proteins such as annexin I and calreticulin [22], [23]. However, PS remains a universal component of the recognition pattern on the surface of apoptotic cells [24], and recent studies have implicated several different macrophage receptors in the process of PS-dependent clearance of cell corpses [25], [26]. In addition, knockout-mouse studies have shown that PS-dependent clearance of apoptotic cells is crucial for the maintenance of tissue homeostasis [27]. Therefore, we hypothesized that coating of SWCNT with PS will interface them with macrophages and stimulate the recognition, tethering and engulfment of nanotubes. Thus, PS-coated SWCNT can be utilized for targeted delivery of specialized cargos - regulators, inhibitors - into macrophages to control their functions including inflammatory responses to SWCNT themselves. Here we report that PS-coated SWCNTs are indeed readily taken up by various classes of phagocytic cells (macrophages, microglia, and dendritic cells). Further, using PS-coated SWCNTs we were able to successfully deliver cytochrome c (cyt c), a pro-apoptotic death signal – and cause apoptosis in macrophages. These studies thus demonstrate that non-covalent modification of SWCNTs with specific phospholipid molecules can be employed for targeted delivery and regulation of professional phagocytes.

## Materials and Methods

### Reagents

1,2-Dioleoyl-*sn*-Glycero-3-Phosphocholine (DOPC), 1,2-Dioleoyl-*sn*-Glycero-3-[Phospho-L-Serine] (Sodium Salt) (DOPS), 16∶0-6∶0 NBD PS, 1-Palmitoyl-2-[6-[(7-nitro-2-1,3-benzoxadiazol-4-yl)amino]dodecanoyl]-*sn*-Glycero-3-Phospho-L-Serine (Ammonium Salt) and 16∶0-6∶0 NBD PC, 1-Palmitoyl-2-[6-[(7-nitro-2-1,3-benzoxadiazol-4-yl)amino]dodecanoyl]-*sn*-Glycero-3-Phospho-choline (Ammonium Salt) were from Avanti Polar Lipids Inc. (Alabaster, AL). HEPES, MgCl_2_, KCl, NaCl, phenylmethylsulfonyl fluoride, glutaraldehyde, osmium tetroxide, potassium ferricyanide, diethylenetriaminepentaacetic acid (DTPA), zymosan and Hoechst 33342 were from Sigma-Aldrich (St. Louis, MO). RPMI, DMEM medium, Ca^2+^+Mg^2+^-free PBS were purchased from Invitrogen Corporation (Grand Island, NY).

### Particles

SWCNT (CNI Inc., Houston, TX) produced by the high pressure CO disproportionation process (HiPco) technique [28], employing CO in a continuous-flow gas phase as the carbon feedstock and Fe(CO)_5_ as the iron-containing catalyst precursor, and purified by acid treatment to remove metal contaminates [29] were used in the study. Chemical analysis trace metal (iron) in SWCNT was performed at the Chemical Exposure and Monitoring Branch (DART/NIOSH, Cincinnati, OH) using nitric acid dissolution and inductively coupled plasma-atomic emission spectrometry (ICP-AES). Analysis revealed that SWCNT comprised of 0.23 weight % iron. For purity assessment of Hipco SWCNT, we used several standard analytical techniques including thermo gravimetric analysis with differential scanning calorimetry (TGA-DSC), thermo-programming oxidation (TPO), and Raman and Near-Infrared (NIR) spectroscopy [30]. Comparative analytical data obtained by TGA-DSC, TPO, NIR and Raman spectroscopy revealed that more than 99% of carbon content in the SWCNT HiPco product was accountable in CNT morphology. Purified suspended HiPco SWCNT [29] were used in the study. SWCNT were routinely tested for bacterial endotoxin (LPS) contamination using the endpoint chromogenic LAL method, as previously described [31]. The mean diameter and surface area of SWCNT was 1–4 nm and 1040 m^2^/g. Surface area was determined by Brunauer, Emmett, and Teller (BET) analysis, and diameter and length was measured by TEM.

The chemical cutting of SWCNT was performed as reported previously [32]. Purified SWCNT were dispersed in 4∶1 mixture of concentrated H_2_SO_4_ and 35% aqueous H_2_O_2_ and sonicated in ultrasonic bath (Branson 1510 Sonifier®, output power of 70 W at 40 KHz) for 24 hrs at 0°C. The dispersion was then heated to 70°C for 10 min for “polishing” the nanotubes. This solution was then diluted 10-fold by deionized water and filtered through PTFE membrane (100 µm pore size). The collected sample was thoroughly washed with deionized water and vacuum dried at 110°C for 30 min. Thus obtained short SWCNT were dispersed in 25 mM HEPES buffer (pH 7.4; containing 150 mM NaCl) by sonication to final concentration 0.5 mg SWCNT/ml.

Transmission electron microscopy was conducted in a FEI-Morgani TEM operated at 80 KV equipped with a soft imaging system charge-coupled device (CCD) camera. TEM samples were prepared by drop casting the solution on copper grid and the excess drawn off with filter paper. The grid was negatively stained with 2% uranyl acetate solution for a minute.

Zeta potential and particle size were determined on the Malvern Zetasizer Nano (Malvern Instruments, Westborough, MA). The analysis was conducted according to standard operating procedure (Particle Technology Labs, Ltd, Downers Grove, IL) for this instrumentation type.

Elemental carbon was analyzed using modified method NIOSH 5040 (NMAM 5040) [33]. After sonication and vortexing, samples (50 µl) were spiked onto 1.45 cm^2^, clean quartz filter punch and placed in Petri dish. The samples were allowed to dry overnight in a dessicator located in the balance room. The quartz punch was then analyzed with the Sunset thermal-optical carbon analyzer (Sunset Laboratory Inc., Tigard, OR).

### Atomic force microscopy (AFM)

After sonication, 20 µL of SWCNT in 25 mM HEPES buffer (pH 7.4) was placed on a freshly cleaved mica which was subsequently rinsed with DI water while being spun at 950 rpm to remove the excess of the buffer solution. The samples were air-dried prior to imaging. AFM images were collected in a tapping mode with a Multimode Nanoscope IIIa microscope (Digital Instruments, Santa Barbara, CA) in air.

### Coating of SWCNT with phospholipids and other cargoes

In all presented experiments, the SWCNT subjected to chemical cutting by H_2_SO_4_ plus H_2_O_2_ have been utilized. The morphology of thus obtained SWCNT preparations was assessed by TEM, SEM as well as AFM. Following the chemical cutting of SWCNT, the nanotubes were used for coating with cargoes (phospholipids, cyt c). Therefore, neither phospholipids nor cyt c were exposed to aggressive environments employed for the SWCNT cutting protocol. SWCNT were sonicated with either 2.5 mM DOPC or 5 mM DOPC: DOPS at the ratio of 1∶1 (3 cycles 30 s), then washed 4 times with 25 mM HEPES, pH 7.4. After each washing, samples were centrifuged at 50,000 g for 30 min at 4°C. To prepare fluorescently labeled nanotubes, SWCNT were sonicated with either PC or PC/PS liposomes containing NBD-PC or NBD-PS (10% of total phospholipids), respectively. For the PS-coated Annexin V treated SWCNT, PS-coated SWCNT were incubated with Annexin V (25 mg/mg SWCNT) in Annexin V binding buffer for 5 min at room temperature and then washed twice with 25 mM HEPES, pH 7.4 to remove non-bound Annexin V. After each washing, PS-coated/Annexin V treated SWCNT were centrifuged at 50,000 g for 15 min. To prepare PS/cyt c/SWCNT, nanoparticles (0.3 mg/ml) were incubated in 25 mM HEPES buffer, pH 7.4 with 50 µM cyt c for 30 min at room temperature. To remove non-bound cyt c, SWCNT were washed twice with 25 mM HEPES buffer pH 7.4 and centrifuged at 50,000 g for 30 min at 4°C. After that, cyt c/SWCNT were sonicated in the presence of 3 mM PC∶PS liposomes (at the ratio of 1∶1) 3 cycles for 30 s and then washed 4 times with 25 mM HEPES. After each washing, samples were centrifuged at 50,000 g for 30 min at 4°C. Coated SWCNT were finally suspended in 25 mM HEPES pH 7.4 (containing a transition metal chelator, DTPA (100 µM) to prevent oxidative damage to lipids and protein) using the same volume as the original suspension. Endotoxin content in SWCNT suspensions and its amounts present in the medium during incubations were approximately 300–500 times lower than those causing stimulation of macrophages.

### Determination of phospholipid content

Phospholipids from coated SWCNT were extracted using Folch procedure [34] and separated by one dimensional HPTLC. Spots of PS or PC were visualized by exposure to iodine vapors and compared with authentic standards. Phospholipid phosphorus was determined using sub-micro method [35].

### Animals

Specific-pathogen-free adult female C57BL/6 mice (7–8 wk) were supplied by Jackson Lab (Bar Harbor, ME) and weighed 20.3±0.2 g at time of use. Animals were individually housed in AAALAC-approved NIOSH animal facilities in microisolator cages for one week prior to use. Autoclaved Beta Chip bedding (Northeastern Products Corp., Warrensburg, NY) was changed weekly. Animals were supplied with water and Harlan Teklad, 7913, NIH-31 Modified Mouse/Rat Diet, Irradiated (Harlan Teklad, Madison, WI) and housed under controlled light, temperature and humidity conditions. Experiments were conducted under a protocol approved by the Animal Care and Use Committee of the NIOSH. Mice were randomized into three experimental groups treated either with non-coated SWCNT, PC-coated SWCNT or PS-coated SWCNT on day 0. Animals were sacrificed on day 1 following exposures.

### Particulate Instillation

Pharyngeal aspiration was used for particulate administration to C57BL/6 mice. Briefly, after anesthization with ketamine and xylazine anesthesia (62.5 and 2.5 mg/kg, respectively), the mouse was placed on a board in a near vertical position. The animal's tongue was extended with lined forceps and a suspension of particulates (50 µl, non-coated SWCNT, PC-coated SWCNT or PS-coated SWCNT at a dose of 40 µg/mouse) was placed in the posterior of pharynx. The tongue was held until the suspension was aspirated into the lungs. All mice in particle and PBS groups survived this exposure procedure. This technique provides good distribution of particles widely disseminated in a peri-bronchiolar pattern within the alveolar region [36]. Animals treated with the particulates recovered easily after anesthesia with no behavioral or negative health outcomes.

### Bronchoalveolar lavage

Mice were weighed and euthanized with intraperitoneal injection of sodium pentobarbital (SPB, Fort Dodge Animal Health, Fort Dodge, Iowa) (>100 mg/kg). The trachea was cannulated with a blunted 22 gauge needle, and BAL was performed using cold sterile Ca^2+^+Mg ^2+^-free PBS at a volume of 0.9 ml for first lavage (kept separate) and 1.0 ml for subsequent lavages. Approximately 5 ml of BAL fluid per mouse was collected and pooled in sterile centrifuge tubes. Pooled BAL cells were washed in Ca^+2^+Mg^+2^-free PBS by alternate centrifugation (800×*g* for 10 min at 4°C) and resuspension.

### Cells

Primary microglia was isolated from brains of postnatal day 5 rats as described [37]. More than 80% of the cell population was represented by microglia as evidenced by immunostaining with the microglia CD11 (mouse monoclonal anti-CD11 antibody were from Novus Biologicals, Inc. (Littleton, Colorado, USA); Alexa Fluor 488 goat anti-mouse secondary antibody were from Molecular Probes (Eugene, Oregon USA).

Mononuclear cells were prepared from buffy coats (Karolinska University Hospital Blood Bank, Stockholm, Sweden) obtained from healthy adult blood donors by density gradient centrifugation using Lymphoprep (Axis-Shield, Oslo, Norway) or Ficoll Paque (Amersham Pharmacia Biotech AB, Uppsala, Sweden), as described previously [38]. These studies were approved by the local ethical committee at Karolinska Institutet, Stockholm. Briefly, cells were washed and resuspended at 5.0×10^6^ cells/ml in RPMI-1640 medium. Monocytes were separated by adhesion to tissue culture plastic for 1 h at 37°C with a 5% CO_2_ atmosphere and non-adherent cells were removed by several washes with PBS. Human monocyte-derived macrophages (HMDMs) were cultured for 3–4 days in RPMI-1640 medium supplemented with 10% heat-inactivated FBS, 2 mM glutamine, 100 U/ml penicillin and 100 µg/ml streptomycin (Gibco Invitrogen Corporation, Paisley, UK), and 50 ng/ml human recombinant M-CSF (50 ng/ml = 7500 IU/ml) (R&D Systems, Abingdon, UK). For isolation of dendritic cells, peripheral blood mononuclear cells were harvested as described above, and washed three times with PBS, followed by resuspension in MACS-buffer (80 µl/10^7^ cells) containing 0.5% BSA, 2 mM EDTA in PBS. Anti-CD14 microbeads (Miltenyi Biotech, Bergisch Gladbach, Germany) were added according to the manufacturer's instructions. After 30 min at 4°C, the CD14-positive cells were separated from the solution by autoMACS (Miltenyi Biotec), and analyzed by flow cytometry (FACSCalibur, Becton Dickinson, Franklin Lakes, NJ) to check for CD14^+^ cell purity. Monocyte-derived dendritic cells (MDDC) were generated essentially as described before [39] by culturing monocytes in RPMI 1640 medium, supplemented with 25 µg/mL gentamicin, 2 mM L-glutamine, 100 IU/ml penicillin, 100 µg/ml streptomycin, 50 µM β-mercaptoethanol, 10% heat-inactivated FCS, and the recombinant cytokines GM-CSF (550 IU/mL), and IL-4 (800 IU/mL), at a density of 4.0×10^5^ cells/ml, at 37°C in a 5% CO_2_ atmosphere. Both cytokines were from Biosource International (Camarillo, CA). After 6–7 days, the cell surface molecules CD1a, CD11c, CD14 and CD83 were analyzed by flow cytometry as described below, to confirm an immature, phagocytosis-competent phenotype with low CD83 expression.

HeLa cells and RAW264.7 macrophages (American Tissue Culture Collections; ATCC) were grown in DMEM supplemented with 10% heat inactivated fetal bovine serum (FBS), 100 units/ml penicillin and 100 µg/ml streptomycin.

SH-SY5Y neuroblastoma cells (ATCC) were kept in DMEM∶F12 (1∶1) supplemented with 2 mM glutamine, 1% non essential amino acids and 15% fetal bovine serum (FBS).

### Cell exposure to particles

Cells (at density of 0.3×10^6^/ml for RAW 264.7 macrophages and HeLa cells, 0.5×10^6^/ml for HMDM and MDDC, 3×10^5^/ml for microglia and SH-SY5Y neuroblastoma cells) were exposed to non-coated SWCNT or PC-coated SWCNT, PS-coated SWCNT (0.02 mg/ml, calculated by concentration of SWCNT, or 0.1 mg/ml for HMDM and MDDC) for 2 h (and 24 h for some experiments using MDDC) in serum-free medium, except for primary human phagocytes which were maintained in cell culture medium supplemented with 10% heat-inactivated serum so that cell viability was not compromised. For the purpose of uniformity we present the condition of exposure to SWCNT using the ratios of SWCNT (µg)/10^6^ cells. The ratios SWCNT to cells were 125–150 µg/10^6^ cells. In the experiments with cyt c/PS-coated SWCNT chloroquine (100 µM) was applied as endosome disruptor and cells were incubated for 15 min at 37°C. Cells were washed once with DMEM medium and then incubated in DMEM medium containing 10% FBS for additional 2 h. At the end pointed incubation, cells were washed with PBS, collected and used for assessment of caspase 3/7 activity and externalized of PS. Cytotoxicity was confirmed using Trypan blue exclusion as well as LDH release (with CytoTox-ONE™ Homogeneous Membrane Integrity Assay (Promega, WI) kit).

### Assessment of cytokines

RAW macrophages were seeded at 2.5×10^5^ cells/well 12 h before treatment. Cells were incubated with 0.25 mg/ml zymosan in the presence or absence of SWCNT, PS-coated SWCNT or PC-coated SWCNT (all at the level of 150 µg/10^6^ cells) in normal culture medium for different time periods. At the end of incubation, the medium was collected and subjected to 50,000 g centrifugation at 4°C for 30 min to remove nanoparticles from the solution. The supernatant was then used to measure cytokines. R&D Quantikine ® immunoassay kit (R&D system Inc. Minneapolis, MN) was used for measurements according to the manufacturer's manual.

Caspase 3/7 activity was measured using Caspase-Glo 3/7 Assay kit (Promega, Madison WI, USA).

PS exposure was determined by fluorescently microscopic detection of annexin V as outlined in the annexin V-FITC apoptosis detection kit (BioVision Research Products, Mountain View, CA). Cells were analyzed under a Nikon ECLIPSE TE 200 fluorescence microscope (Tokyo, Japan) equipped with a digital Hamamatsu CCD camera (C4742-95-12NBR) using the MetaImaging Series™ software version 4.6 (Universal Imaging Corp., Downingtown, PA). A minimum of 300 cells were analyzed per experimental condition.

### Flow cytometry

Fluorescence intensity of HMDM or MDDC incubated in the presence or absence of NBD-labeled PC/PS-coated SWCNT was measured with a FACScan flow cytometer (Becton Dickinson, San Jose, CA) equipped with a 488 nm argon laser. Ten thousand events were gated for live cells based on forward and side scatter characteristics were collected for each sample and data were analyzed using CellQuestPro software (Becton Dickinson). For monitoring of DC phenotype, cells were labeled with fluorescent phycoerythrin (PE)-conjugated monoclonal antibodies (mAbs) specific for CD1a (Coulter Corporation, Hialeah, FL) and CD11c (Becton Dickinson); and with fluorescein isothiocyanate (FITC)-conjugated mouse mAbs specific for CD83 and CD14 (Becton Dickinson) according to the manufacturer's instructions. Control samples were labeled with isotype-matched antibodies conjugated with the same fluorochrome. Fluorescence was measured with a FACSCalibur flow cytometer (Becton Dickinson) and data were analyzed using CellQuestPro.

### Fluorescent microscopy

Cells were seeded on cover slides 18 h before treatment, 20 µg/ml of various functionalized SWCNT were added and incubated in DMEM with no phenol red at 37°C for 2 h. At the end of incubation cells were gently washed 3 times with PBS, fixed by 2.5% paraformaldehyde at RT for 5 min and examined under a Nikon ECLIPSE TE 200 fluorescence microscope (Tokyo, Japan) equipped with a digital Hamamatsu charge-coupled device camera (C4742–95-12NBR) and analyzed using the MetaImaging Series™ software version 4.6 (Universal Imaging Corp., Downingtown, PA).

### Confocal microscopy

RAW 264.7 macrophages were seeded on LabTek chamber slides at a density of 5×10^5^ cells per well the previous day. The normal DMEM medium was replaced with phenol red free RPMI 1640 medium the next day and incubated with LysoTracker Red- DND99 (50 nM) for 1 h to label lysosomes. Prior to incubation with LysoTracker Red, RAW 264.7 macrophages (5×10^5^ cells per well) were incubated with cocktail of endocytosis inhibitors containing a mixture of nystatin (25 µg/ml), genistein (200 µM), chlorpromazine (6 µg/ml) and brefeldin A (10 µg/ml) for 30 min as described [40]. Cells were then washed twice with PBS followed by addition of various functionalized SWCNT (150 µg/10^6^ cells). The cells were incubated with particles for 5 minutes for the purpose of confocal microscopy. Cells were then washed three times with PBS and fixed using 2% paraformaldehyde. Nuclei were stained with Hoechst 33342. Cells were imaged using Olympus Fluoview 1000 confocal microscope.

Following incubation for 2 or 24 h with NBD-labeled SWCNT, MDDCs were fixed in 4% formaldehyde for 15 min. Staining of cell membranes was carried out using anti-HLA-DR mAb (Becton Dickinson). A secondary goat anti-mouse mAb labeled with Alexa Fluor 546 (Molecular Probes, Eugene, OR) was used for detection. Slides were mounted with anti-fading Vectashield mounting medium (Vector laboratories, Burlingame, CA). Fluorescent images were acquired on a confocal laser-scanning microscope (TCS SP2; Leica Microsystems, Mannheim, Germany) equipped with one argon and two HeNe lasers. NBD was excited with a 488-nm laser line detecting light in the wavelength region of 560–700 nm. Alexa 546 was excited by a 543-nm laser line with detection of light in the region of 560–700 nm.

### Scanning Electron Microscopy

Macrophages fixed in cold 2.5% glutaraldehyde were rinsed in PBS, post-fixed in 1% Osmium Tetroxide (Electron Microscopy Sciences, Hatfield, PA) with 0.1% potassium ferricyanide (Fisher Scientific, Pittsburgh, PA) dehydrated through a graded series of ethanol, from 30 to 100%, and then critical point dried in a critical point dryer, Emscope CPD, (EMScope Lab, Ashford, Great Britain). Following critical point drying, the samples were attached to aluminum SEM specimen mounting stubs (Electron Microscopy Sciences) and then sputter coated with a gold palladium alloy (Sputter Coater 108 Auto, Cressington Scientific Instruments, Cranberry Township, PA). Following processing, samples were analyzed using a JEM 6330F microscope (JEOL, Peabody, MA).

### Transmission Electron Microscopy

Macrophages were fixed in 2.5% glutaraldehyde for 1 h, pelleted, and re-suspended in 3% gelatin in PBS, olidified at 4°C, and then re-fixed for 15 min. Pellets were washed 3 times in PBS and then postfixed in 1% OsO_4_ and 1% K_3_Fe(CN)_6_ for 1 hour. After 3 PBS washes, the pellet was dehydrated through a graded series of 30% to 100% ethanol then incubated in Polybed 812 epoxy resin (Polysciences, Warrington, PA) for 1 h. After several changes of 100% resin over 24 h, pellet was cured at 37°C overnight with additional hardening at 65°C for 48 h. Ultrathin (60 nm) sections were collected on 200 mesh grids and stained with 2% uranyl acetate in 50% methanol for 10 min followed by 1% lead citrate for 7 min. Sections were observed on a JEM 1210 electron microscope (JEOL, Peabody, MA) at 80 kV.

### Statistics

The results are presented as mean±s.d. values from at least three experiments, and statistical analyses were performed by either paired/unpaired Student's t-test or one-way ANOVA. The statistical significance of differences was set at *p*< 0.05.

## Results

### Physico-chemical characterization of functionalized SWCNT

**Figure 1:**
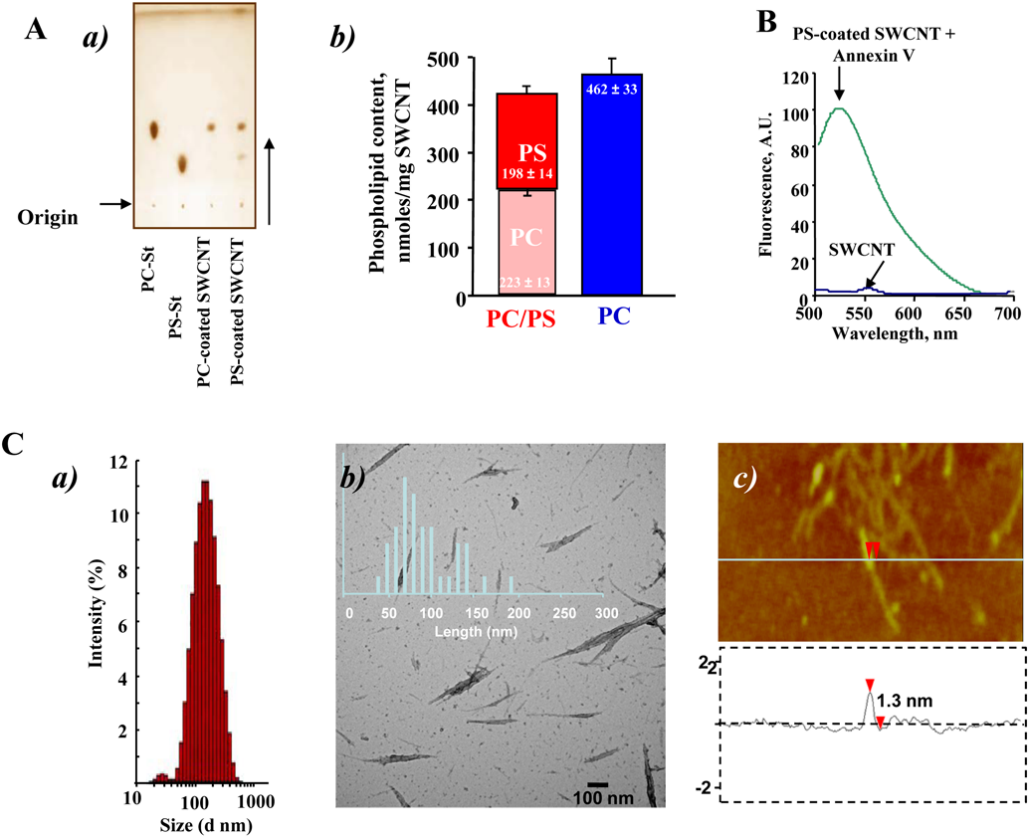
Physico-chemical characterization of SWCNT. A. Evaluation of phospholipid content of PC-coated or PS-coated SWCNT. a) Typical one-dimensional HPTLC of phospholipids extracted from PC-coated or PS-coated SWCNT. b) Phospholipid content of phospholipid-coated SWCNT. B. Typical fluorescence spectrum of Annexin V bound to PS-coated SWCNT. Note that a robust fluorescence response (Annexin V-FITC binding) was recorded from PS-coated but not from non-coated or PC-coated SWCNT. C. Physico-chemical characterization of bare SWCNT. a) Typical histograms of size distribution assessed by dynamic light scattering; b) AFM images of bare SWCNT deposited on mica substrates; c) The height cross section analysis of bare SWCNT.

We prepared SWCNT coated with DOPC, as a control (PC-coated SWCNT), or a mixture of DOPS, plus PC (PS-coated SWCNT) by incubating nanotubes in the presence of liposomes containing these lipids (PC or a mixture of PS plus PC at a molar ratio of 1∶1). Successful integration and the content of PS and PC associated with SWCNT were confirmed by direct assessment of the phosphorus content after HPTLC separation of phospholipids (Fig. 1Aa). We found that PC-coated SWCNT contained 462±33 nmol PC/mg SWCNT. Analysis of PS-coated SWCNT revealed that amounts of integrated PS and PC on nanotubes were approximately similar: 198±14 and 223±13 nmol of phospholipids/mg SWCNT, respectively (Fig. 1Ab). Additionally, the presence of PS on the surface of SWCNT was verified using FITC-conjugated PS-specific protein, Annexin V, by measuring its characteristic fluorescence (Fig 1B). After treatment of PS-coated SWCNT with FITC-conjugated Annexin V, the appearance of a robust fluorescence response was observed.

Analysis performed by NMAN 5040 revealed that purified SWCNT were comprised of 99.7 wt% elemental carbon. Determinations of carbon content for non-coated, PS-coated, PC-coated and cyt c/PS-coated SWCNT showed that non-covalent functionalization of SWCNT with phospholipids and protein (cyt c) expectedly resulted in increased carbon content in SWCNT suspensions. Based on our HPTLC-based determinations of phospholipid content of PC- and PS-coated SWCNT, the expected content of elemental carbon in the suspensions of these coated SWCNT should be 81 and 61 ng/µl, respectively. Direct estimates of elemental carbon content were 75 and 59 ng/µl for SWCNT functionalized with PS and PC (Table S1). These numbers correspond to 87% and 97% of theoretically calculated content of the two phospholipids, respectively.

There was essentially no difference in the organization and structure of SWCNT between non-coated samples and PS- or PC-coated samples. Assessments of size distribution by dynamic light scattering (DLS) showed that SWCNT (Fig. 1Ca), PS-coated SWCNT and PC-coated SWCNT samples in suspensions had very similar particle sizes in the range between 130–180 nm (Table S1). Thus, SWCNTs were similarly dispersed in all formulations used. Statistical analysis of isolated SWCNTs measured by TEM revealed length distribution with a mean length of 90±35 nm (Fig. 1Cb). DLS determined the average hydrodynamic diameter of the same sample as ≈135 nm. Average particle sizes from DLS measurements for all samples are summarized in Table S1. Small increases in particle size indicate that no significant aggregation of SWCNTs takes place in solution. Moreover, it is known that in microscopy, assessments may be length-biased because they do not include nanotubes that cross the edge of the micrograph and those that aggregate [41]. In fact, longer nanotubes are ignored, because they are more likely to reach out of view and aggregate with each other.

Evaluations of zeta potentials for SWCNT, PC-coated SWCNT, PS-coated SWCNT, PS-NBD-coated SWCNT, PC-NBD-coated SWCNT, and SWCNT containing cyt c showed that they ranged from −40 to −50 mV (Supplemental Table 1). Coating of SWCNT with phospholipids caused a slight increase in the negative values of zeta potentials which, however, was not significant between PC-coated SWCNT and PS-coated SWCNT. Overall, the estimated negative values of zeta potentials correspond with significant stability of dispersed non-coated and phospholipids-coated SWCNTs in aqueous suspensions [42], [43].

Transmission electron microscopy (TEM) of negatively stained non-coated, PS-coated, and PC-coated SWCNT showed that they had typical fibrous morphology and were represented mostly by single nanotubes, as well as by ropes of nanotubes (Fig. 1Cb) and entangled “bird's nest”-like aggregates. (Figure S1A). Calculated percentage contributions of each of these “morphologies” in the formulations used were 70, 25 and 5% for single SWCNT, bundles and “bird's-nest”-like aggregates, respectively (Fig 1Cb insert). PS-coated and PC-coated SWCNT had similar morphology as non-coated SWCNT. Moreover, a thin layer of phospholipids (PS/PC) (stained) is clearly visible on the sidewalls of SWCNT by TEM (Figure S1B).

### Cytotoxicity of SWCNT

Both Trypan Blue exclusion test and LDH release assay demonstrated that neither non-coated SWCNT nor phospholipids-coated SWCNT induced any cytotoxic effects in RAW 264.7 macrophages after 4 hrs of co-incubation (Figure S2). Moreover, no impairment of viability was seen at 4 and 24 h of incubation of SWCNT with primary human macrophages, using the Trypan blue assay and the Hoechst 33342 staining method for visualization of cell nuclei (data not shown).

### Scanning electron microscopy (SEM) imaging of SWCNT

**Figure 2:**
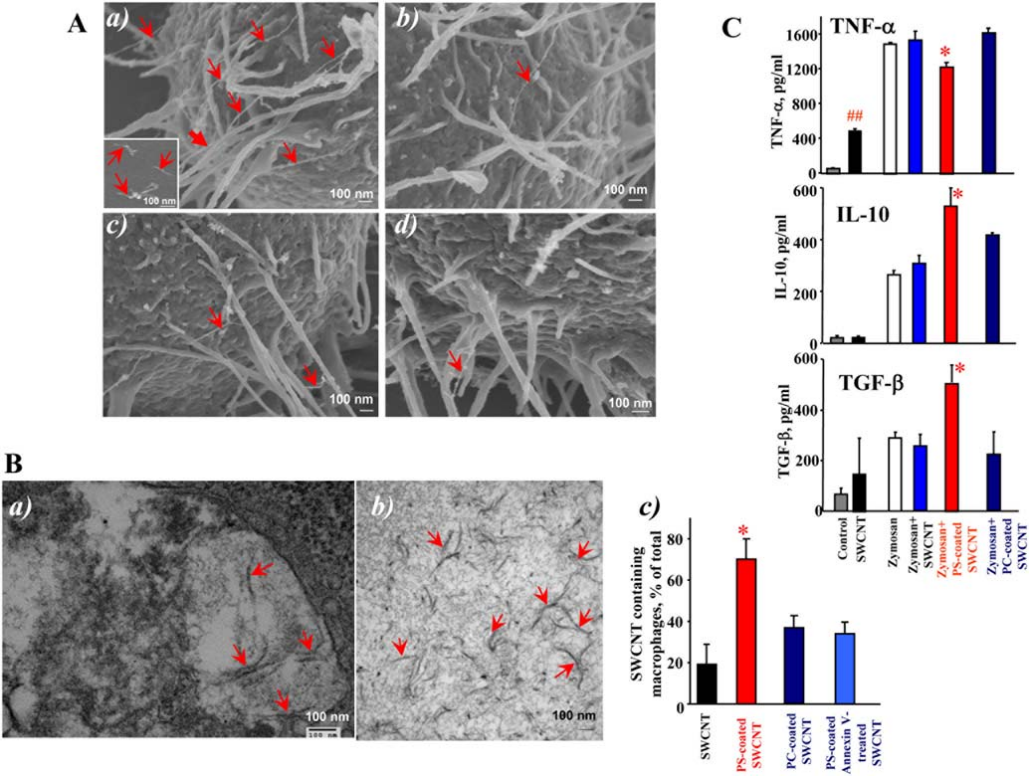
SWCNT functionalized with PS but not with PC engulfed by murine RAW264.7 macrophages. A. Scanning electron micrographs of RAW264.7 macrophages treated with SWCNT *in vitro*. RAW264.7 macrophages (0.3×10^6^ cells/ml) were incubated for 2 h with non-coated, PC- or PS-coated SWCNT. At the end of incubation macrophages were washed and fixed for SEM. a) Recognition of PS-coated SWCNT by RAW264.7 macrophages; (Note: The red arrows in all the electron micrographs in figure 2 point SWCNT. Sub-panel A-a represents scanning electron micrograph of SWCNT alone.) b) PC-coated SWCNT, c) non-coated, d) PS-coated Annexin V treated SWCNT are poorly recognized by macrophages. A total of 150 cells from each sample type were analyzed by SEM. B. Transmission electron micrographs of RAW264.7 macrophages treated with SWCNT *in vitro*. RAW264.7 macrophages (0.3×10^6^ cells/ml) were incubated for 2 h with non-coated, PC- or PS-coated SWCNT. At the end of incubation macrophages were washed and fixed for TEM. a) A high-power image of the macrophage with engulfed PS-coated SWCNT. b) A typical TEM image of PS-coated SWCNT. Samples of SWCNT for TEM imaging were processed in a fashion similar to that of cells for electron microscopy. Arrows indicate SWCNT. Representative TEM images are presented. c) Quantitative assessments of SWCNT phagocytosis by RAW 264.7 macrophages. A total of 150 cells from each sample type were analyzed by TEM. Data are mean±S.D., n = 3, *p<0.05, PS-coated SWCNT vs SWCNT, PC-coated SWCNT and PS-coated Annexin V-treated. C. Effect of SWCNT on cytokine production by zymosan-stimulated RAW 264.7 macrophages. Macrophages were seeded at 2.5×10^5^ cells/well in 48 well plates and co-incubated with zymosan (0.25 mg/ml) in the presence of non-coated, PC-coated and PS coated SWCNT (150 µg/10^6^ cells). At the end of 2 hr incubation, TNF-α was measured in the medium. IL-10 and TGF-β were measured after 4 hr incubation. The cytokines were measured using R&D Quantikine ® immunoassay kit. Data are mean±s.d., n = 3. ^##^p<0.05, SWCNT vs control. *p<0.05, PS-coated plus zymosan *vs* SWCNT plus zymozan and PC-coated plus zymosan.

To investigate interactions of SWCNT with the surface of macrophages, we performed SEM imaging (Fig. 2A). We found that PS-coated SWCNT were actively recognized and tethered to the surface of RAW264.7 macrophages. At higher magnifications (Fig. 2Aa), we were able to detect the presence of SWCNT fibers on the surface of macrophages. Numerous filopodia – budding off the macrophages surface – associated with their major functions such as phagocytosis and substrate adhesion were observed in RAW264.7 macrophages exposed to PS-coated SWCNT. In contrast, non-coated (Fig. 2Ab), PC-coated (Fig. 2Ac) and PS-coated Annexin V treated (Fig. 2Ad) SWCNT were ingested to a lesser extent by macrophages, and the presence of these types of SWCNT on the surface of RAW264.7 macrophages was observed much less frequently.

### Transmission electron microscopy (TEM) of SWCNT

TEM evaluations of the uptake of SWCNT showed that RAW264.7 macrophages readily phagocytozed PS-coated SWCNT (Fig. 2Ba) while a significantly less pronounced engulfment of PC-coated and non-coated SWCNT was documented (Fig. 2Bc). In addition, macrophages co-incubated with PS-coated SWCNT had more endocytotic vesicles with entrapped nanoparticles compared to macrophages incubated in the presence of PC-coated or non-coated SWCNT (data not shown). At higher magnifications (Fig. 2Ba), PS-coated SWCNT fibers within phagosomes could be seen. To distinguish SWCNT from autophagic bodies inside of phago-lysosomes we used a high magnification TEM imaging (100,000×). Further, in order to better identify nanoparticles in cells we performed TEM imaging of nanotubes alone which were processed in a fashion similar to that of cells for electron microscopy. SWCNT alone appear as “bamboo shoots” and resemble SWCNT that have been engulfed by RAW264.7 macrophages (Fig. 2Bab). Markedly less of “bamboo shoot”-like material was detected in RAW264.7 macrophages exposed to PC-coated or non–coated SWCNT (Fig. 2Bc). Quantitative assessments of the number of nanotubes present in an endosome within a 60 nm ultrafine section of cells could be complicated by the fact that the same SWCNT could be cut 1, 2 or more times, especially SWCNT bundles or “bird's-nest”-like aggregates. Therefore, we presented the data as a plot of percentage of active phagocytes in analyzed samples. We considered as active macrophages those that engulfed at least one nanoparticle. We found that amongst the various treatment groups, 70% macrophages treated with PS-coated SWCNT showed active phagocytosis with nanoparticles inside, which was approximately twice the phagocytosis-positive population of cells co-incubated with PC-coated SWCNT. Annexin V that masks PS [44] and affects its recognition [45], effectively blocked the recognition by 50% as indicated by a lower cell population with active phagocytes (Fig. 2Bc). Notably, these TEM-based evaluations of phagocytotic activity towards SWCNT were in good agreement with independent assessments performed by confocal microscopy of NBD-labeled phospholipid-coated SWCNT (see below).

### Uptake of SWCNT functionalized with fluorescently labeled phospholipids

**Figure 3:**
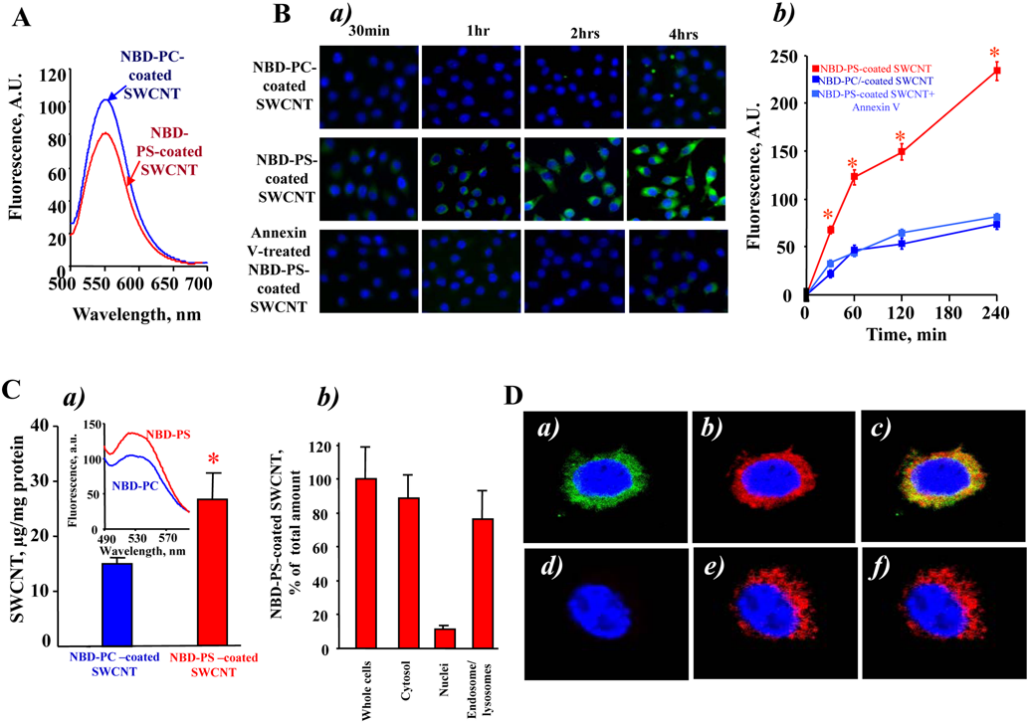
*In vitro* assessment of uptake of NBD-PS-coated or NBD-PC-coated SWCNT by RAW264.7 macrophages. A. Typical fluorescence spectra obtained from NBD-PC- and NBD-PS-coated SWCNT. B. Time-dependent uptake of NBD-PS-coated but not NBD-PC-coated SWCNT. a) RAW264.7 macrophages (0.3×10^6^ cells/ml) were incubated for up to 4 hrs with NBD-PC- or NBD-PS-coated SWCNT. Annexin V prevents engulfment of NBD-PS-coated SWCNT by RAW264.7 macrophages. Overlapped blue and green fluorescence images are presented. b) Quantitative evaluation of cell number with engulfed SWCNT. Data are mean±s.d., n = 3. *p<0.05, NBD-PS-coated *vs* NBD-PC-coated SWCNT and NBD-PS-coated Annexin-V treated SWCNT. C. Assessment of NBD-phospholipid-coated SWCNT in whole cells and subcellular fractions isolated from RAW264.7 macrophages. a) Uptake of PS-coated and PC-coated SWCNT by RAW264.7 macrophages. Data are mean±S.D., n = 3, *p<0.05, NBD-PS-coated SWCNT vs NBD-PC coated SWCNT. Inset: typical fluorescence spectra obtained from endosomal/lysosomal fraction isolated from RAW264.7 macrophages. b) Intracellular localization of PS-coated SWCNT in RAW264.7 macrophages. Macrophages were incubated with PC-coated or PS-coated SWCNT for 15 min at 37°C. At the end of incubation, subcellular fractions were isolated and examined for the presence of NBD fluorescence. D. Typical confocal microscopy images of RAW 264.7 macrophages with NBD-PS-coated SWCNT. RAW macrophages were treated with NBD-PS-coated SWCNT in the presence of Lyso-Tracker Red for 5 min at 37°C (a,b,c). In the experiments with inhibitors of endocytosis, macrophages were pretreated with a mixture containing nystatin (25 µg/ml), genistein (200 µM), chlorpromazine (6 µg/ml) and brefeldin A (10 µg/ml) for 30 min prior to incubation with NBD-PS-coated SWCNT (d, e, f). a and d - green fluorescence is from NBD-phospholipid coated SWCNT; b and e - red fluorescence is from Lyso-Tracker Red, c and f - overlay of green and red fluorescence.

To more quantitatively characterize uptake of phospholipid-coated SWCNT by macrophages we utilized fluorescently labeled PS, NBD-PS, for coating SWCNT; NBD-PC was utilized in control experiments. After extensive washings, SWCNT incubated with NBD-PS or NBD-PC displayed characteristic fluorescence spectra confirming that the coating with phospholipids was successful (Fig. 3A). We studied the time-course of uptake of NBD-PC-coated SWCNT, NBD-PS-coated-SWCNT and NBD-PS-coated and Annexin V treated SWCNT by RAW264.7 macrophages. To this end, we co-incubated RAW 264.7 macrophages with either NBD-PC- or NBD-PS coated nanoparticles for up to 4 h (Fig. 3Ba). At this time point, viability test using Trypan Blue exclusion revealed only 5.5±0.9% (n = 3) dead cells. Uptake of NBD-PS-coated nanoparticles was time-dependent. NBD-PS fluorescence was already detectable from cells after 30 min and increased in the course of incubation. Ingestion of NBD-PS-coated nanoparticles was approximately 3-fold higher than that of PC-coated SWCNT. We observed a robust and time-dependent increase in the fluorescence response from RAW 264.7 macrophages exposed to PS-coated SWCNT which was not saturable over 4 hrs of incubation. In contrast, a much weaker and saturable (after 1 hr) fluorescence was detectable from RAW 264.7 macrophages treated with PC-coated SWCNT or PS-coated SWCNT in the presence of Annexin V. Quantitatively, these results are summarized in Fig. 3Bb where fluorescence from NBD-phospholipid containing macrophages is shown. Clearly, PS coated SWCNT were specifically and effectively recognized and taken up by RAW264.7 macrophages. In addition, we performed experiments with primary human monocyte-derived phagocytes at two time-points, 2 h and 24 h, and the preferential ingestion of PS-coated SWCNT versus PC-coated SWCNT was evident also at the later time-point, thus arguing against a non-selective mode of uptake of the functionalized SWCNT (see below).

### Intracellular localization of PS-coated SWCNT

Visually, we observed accumulation of “black” (SWCNT-containing) pellets after sedimentation of cells. To quantitatively characterize this effect, we performed measurements of optical density of cell suspensions incubated with PS-coated SWCNT and PC-coated SWCNT. A broad absorbance of SWCNT with characteristic maxima at 1050–1060 and 1260–1270 nm was detected. The intensity of absorbance at 1050–1060 nm was 1.3-fold higher in PS-coated SWCNT than in PC-coated SWCNT samples. We further used fluorescently-labeled phospholipids to characterize the presence of phospholipid-coated SWCNT in cells. We found that NBD-PS coated SWCNT co-incubated with RAW 264.7 macrophages yielded a higher fluorescence response from cell suspensions than NBD-PC coated SWCNT (Fig. 3Ca). Further, the majority of fluorescence was associated with the cytosolic fraction (containing endo-lysosomal vesicles) and was minimal in the fraction of nuclei and cell debris (Fig. 3Cb). We isolated the endo-lysosomal fraction from RAW 264.7 macrophages loaded with NBD-PS-coated SWCNT and found that 77±18% (n = 3) of the fluorescence response was associated with this fraction. This is in line with the recent demonstration of deposition of SWCNT in endo-lysosomal compartments of macrophages upon prolonged incubation [46].

**Figure 4:**
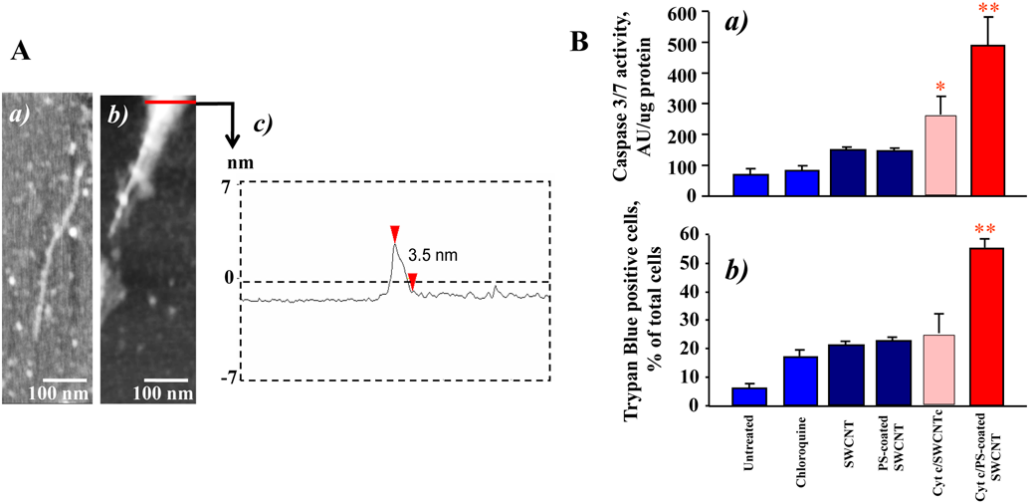
PS-coated SWCNT effectively bind cyt c, deliver it into RAW264.7 macrophages, and activate apoptotic pathways (caspase 3/7), and cell death. A. AFM images of various SWCNT samples deposited on mica substrates: a) SWCNT with cyt c; b) SWCNT with cyt c and PS/PC. c) The height cross section of the functionalized SWCNT in image b. B. Cyt c delivered into macrophages by PS-coated SWCNT activates caspase 3/7 (a) and increases the number of Trypan Blue positive cells (b). SWCNT were treated with 50 µM cyt c, washed twice and then were coated with PS and PC alone. Cells were incubated with protein/lipid/SWCNT conjugates in the presence of 100 µM chloroquine to trigger endosomal rupture. Data are normalized versus SWCNT/chloroquine. Note that under experimental conditions used chloroquine alone (100 µM, 15 min incubation) did not induce any significant activation of caspase 3/7. The data represent mean±s.d (standard deviation), n = 5, *p<0.05, PS-coated plus cyt c *vs* PS-coated SWCNT, non-coated SWCNT plus cyt c and SWCNT alone.

Additionally, to prove co-localization of SWCNT with phagolysosomes we used confocal microscopy. To this end, we treated RAW 264.7 macrophages (5×10^5^ cells/well) with either NBD-PS-coated SWCNT or NBD-PC-coated SWCNT (150 µg/10^6^ cells). We found that NBD-PS-coated SWCNT were readily taken up by macrophages. As shown in Fig. 3Da,b,c, a robust intracellular fluorescence of NBD-PS (but not of NBD-PC) was co-localized, at least in part, with a lysosomal marker, Lyso-Tracker Red. Moreover, after pre-treatment of macrophages with the cocktail of inhibitors of endocytosis (nystatin (25 µg/ml), genistein (200 µM), chlorpromazine (6 µg/ml) and brefeldin A (10 µg/ml)), the intensity of intracellular NBD-PS fluorescence was drastically reduced (Fig. 3Dd,e,f). No fluorescence was detected after treatment of macrophages with NBD-PC-coated SWCNT (data not shown). This suggests that NBD-PS-coated SWCNT were effectively taken up by macrophages through an endocytosis-mediated process resulting in their significant accumulation in endo-lysosomes.

### Effect of SWCNT on cytokine release by RAW264.7 macrophages

Apoptotic cells with externalized PS quench the production and release of pro-inflammatory cytokines by macrophages [15], [19]. To assess whether PS-coated SWCNT display a similar effect, we employed a standard stimulation of TNF-α formation in RAW264.7 macrophages by zymosan [47], [48] and evaluated the effects of SWCNT. PS-coated SWCNT were more potent in inhibiting TNF-α production by stimulated macrophages than PC-coated or non-coated SWCNT (Fig. 2C). Thus, uptake of PS-SWCNT is accompanied by typical PS-dependent suppression of the pro-inflammatory macrophage response. It should be noted that macrophages stimulated with SWCNT alone - in the absence of zymosan - showed a significant increase in the TNF-α production up to 497±24 pg/ml vs. 40±10 (^##^ P<0.05) in the control. Notably, in PS-coated SWCNT, TNF-α levels dropped to 368±33 pg/ml. No significant changes in the content of TNF-α was found after SWCNT coating with PC (445±21 pg/ml). In contrast, the production of anti-inflammatory cytokines is known to be stimulated by PS-dependent pathways [16]. Stimulation of RAW264.7 macrophages by zymosan resulted in the accumulation of TGF-β and IL-10 at 4 hrs after the challenge. The production of anti-inflammatory IL-10 and TGF-β by zymosan-stimulated macrophages subsequently treated with PS-coated-SWCNT increased 1.5- and 2-fold respectively, compared with the effect of zymosan alone (Fig. 2C). This enhancement of IL-10 and TGF-β production was not observed after exposure of zymosan-stimulated RAW264.7 macrophages to either non-coated SWCNT or PC-coated SWCNT.

### PS-coated SWCNT deliver cyt c into RAW264.7 macrophages

We further determined whether PS-coated SWCNT could be employed for the delivery of physiologically active agents to macrophages. Because cyt c released from mitochondria into the cytosol acts as an effective activator of caspases and a death-signal [49] we chose to use it as a cargo. Given that positively charged cyt c readily interacts with negatively charged surfaces we assumed that SWCNT coated with anionic PS would effectively bind to cyt c. Indeed, the binding of cyt c to PS-SWNT was confirmed by direct measurements of specific cyt c absorbance as well as by atomic force microscopy (AFM). Section analysis of bare SWCNT (Fig. 1Cc) demonstrated that the diameter of non-coated SWCNT was 1.3 nm (typically these SWCNTs have diameters in the range of 0.6–1.5 nm [50], [51]. Figure 4Aa shows AFM image of cyt c adsorbed on SWCNT sidewalls. AFM image revealed the presence of globular structures of cyt c on the surface and in the contact with SWCNTs (measured height ∼1 nm). This image is similar to previously reported [6] picture of SWCNTs functionalized with proteins and in particularly with cyt c. AFM of SWCNT functionalized with PS/PC and cyt c displayed a significantly different image (Fig. 4Ab), where distinct PS/PC molecules were not seen; rather a layer of PS/PC was spread on the SWCNT surface thereby demonstrating lipid adsorption onto SWCNTs. Such an event is likely due to a greater retention of PS/PC aggregation, resulting into multiple PS/PC layers over SWCNTs, while individual globular structures of cyt c are still clearly visible on the nanotube sidewalls. The height cross section of the SWCNT functionalized with PS/PC and cyt c (Fig. 4Ac) shows that the diameter of the structures is 3.5 nm which is significantly larger than bare SWCNT.

Importantly, cyt c-loaded PS-SWCNT caused a marked increase of caspase activity (Fig. 4Ba) and PS externalization (Figure S3) in RAW264.7 macrophages upon co-treatment with a disruptor of endosomes, chloroquine. This is in line with previous reports where acid-oxidized SWCNT were utilized for delivery of cyt c into cancer cell lines [6]. PC-coated or non-coated SWCNT pre-incubated with cyt c were ineffective in inducing caspase-3 activation in macrophages. In addition, we found that delivery of cyt c into macrophages resulted in significantly increased numbers of Trypan Blue positive cells (Fig. 4Bb) compared to cells treated with PS-SWCNT with no cyt c attached. Thus, PS-coating makes PS-SWCNT loaded with a protein cargo recognizable, facilitates their uptake by macrophages, and could hence be envisaged as a promising tool for targeted delivery of regulators of macrophage activity/survival.

### Recognition of PS-coated SWCNT by microglia, human monocyte-derived macrophages (HMDM) and human monocyte-derived dendritic cells (MDDC)

**Figure 5:**
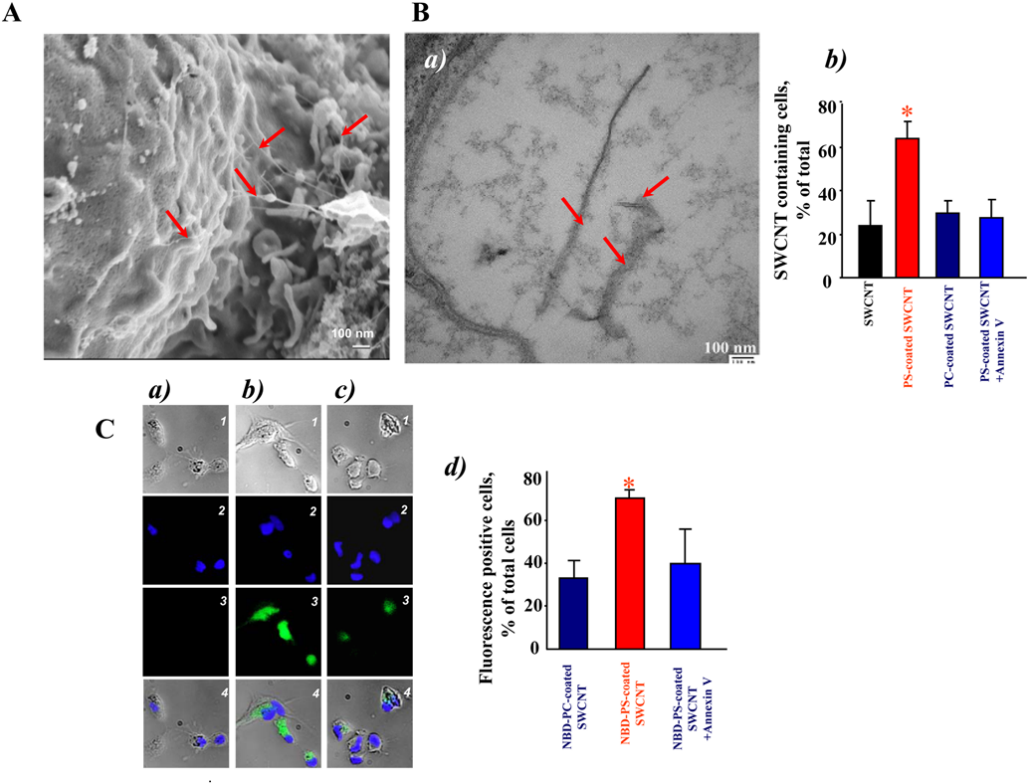
Primary rat microglia recognized SWCNT functionalized with PS but not with PC. A. Scanning electron micrographs of microglia treated with PS-coated SWCNT *in vitro*. Microglia (1.5×10^5^ cells/ml) were incubated for 2 h with PC- or PS-coated SWCNT. At the end of incubation macrophages were washed and fixed for SEM. (Note: The red arrows in all the electron micrographs in figure 5 point SWCNT). B. Transmission electron micrographs of primary microglia exposed to SWCNT *in vitro*. Microglia (1.5×10^5^ cells/ml) were incubated with PC- or PS-coated SWCNT. At the end of incubation microglia was washed and fixed for TEM. a) Microglia exposed to PS-coated SWCNT; b) Quantitative assessments of SWCNT phagocytosis by RAW 264.7 macrophages. A total of 60 cells from each sample type were analyzed by TEM. Data are mean±S.D., n = 3, *p<0.05, PS-coated SWCNT vs SWCNT, PC coated SWCNT and PS-coated Annexin V–treated SWCNT. C. *In vitro* assessment of uptake of SWCNT coated with NBD-PS or NBD-PC by microglia. Microglia (1.5×10^5^ cells/ml) were incubated with NBD-PC- (a), NBD-PS-coated SWCNT (b) or NBD-PS-coated/Annexin V treated SWCNT (c). 1 – bright field image; 2 – blue fluorescence image, Hoechst 33342; 3 – green fluorescence image, NBD-labeled phospholipids; 4 – overlap of blue and green fluorescence images with image under bright field. d) Annexin V treatment of NBD-PS-coated SWCNT prevents their engulfment by microglia. Quantitative evaluation of cell number with engulfed SWCNT. Data are mean±s.d., n = 4. *p<0.05, NBD-PS-coated SWCNT vs NBD-PC-coated SWCNT and NBD-PS-coated Annexin V treated SWCNT.

To determine whether PS-coated SWCNT can be recognized and taken up by other types of professional phagocytes, we performed experiments using microglia from rat brain. The experiments using SEM (Fig. 5A) and TEM (Fig. 5Ba) demonstrated that, similarly to RAW264.7 macrophages, microglia effectively engulfed PS-coated (Fig. 5Bb), but not PC-coated or non-coated SWCNT (data not shown). Essentially in samples in which PS-coated SWCNT were treated with Annexin V the number of SWCNT positive microglial cells was not different from SWCNT controls or from PC-coated SWCNT (Fig. 5Bb). This was confirmed by quantitative assessments of NBD-PS-SWCNT uptake by fluorescence microscopy (Fig. 5C).

**Figure 6:**
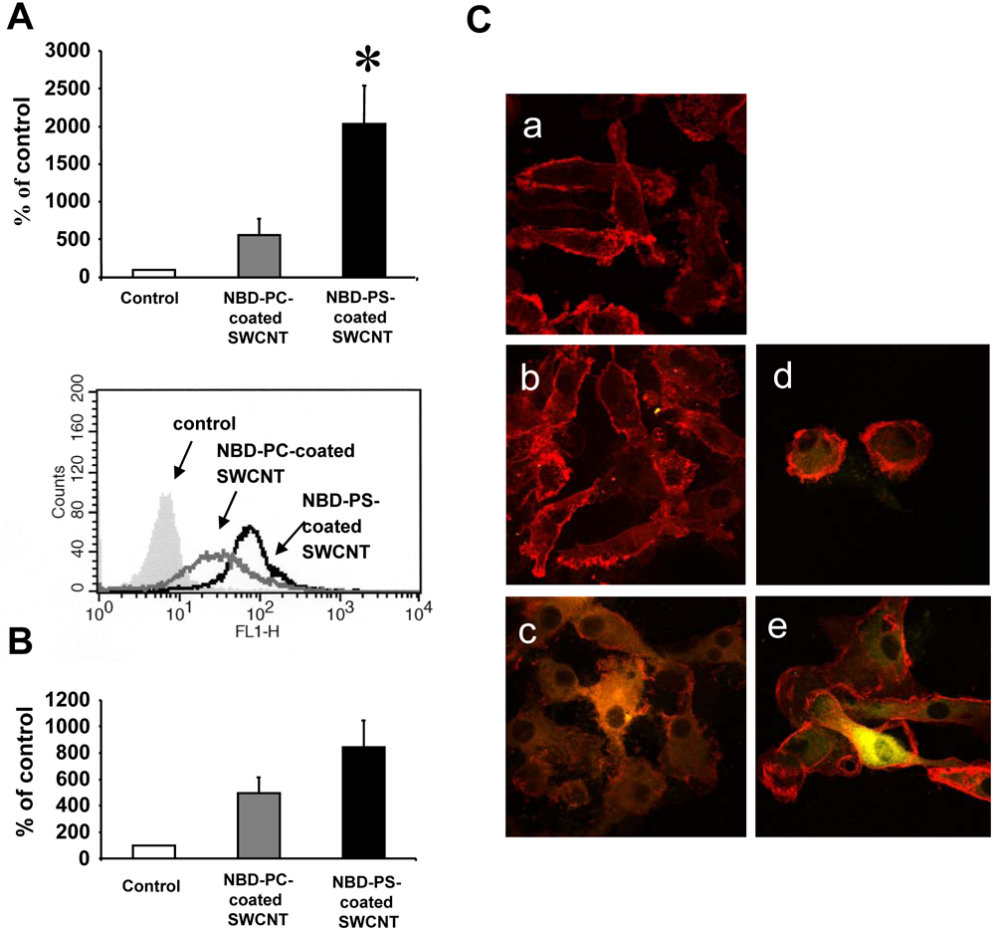
Primary human monocyte-derived macrophages and dendritic cells recognized SWCNT functionalized with PS but not with PC. A. M-CSF-activated HMDM (0.5×10^6^ cells/ml) were incubated with NBD-PC- or NBD-PS-coated SWCNT (100 µg/ml) for 2 h and then subjected to flow cytometric evaluation of uptake of nanotubes. Macrophages incubated without SWCNT were included as an autofluorescence background control, and values are reported as percentage of background control. Representative histograms are shown below the bar graph. PS-coated SWCNT were observed to be taken up by HMDM to a higher degree than PC-coated SWCNT (p<0.002) (n = 4). B. MDDC (0.5×10^6^ cells/ml) exposed to NBD-PC- or NBD-PS-coated SWCNT (100 µg/ml) for 24 h at 37°C were assessed by flow cytometry. Data are reported as above. A tendency toward higher degree of uptake of PS-coated SWCNT was observed compared to PC-coated SWCNT in these cells (*p*<0.06) (n = 3), was seen for these cells. Similar results were obtained when uptake was monitored at 2 h (data not shown). C. Confocal microscopic imaging of MDDC incubated in the absence of SWCNT (a), or in the presence of NBD-PC-coated SWCNT for 2 h (b) or 24 h (d), or NBD-PS-coated SWCNT for 2 h (c) or 24 h (e), respectively. Counterstaining with antibodies to HLA-DR (red) was performed to visualize the plasma membrane of dendritic cells and the yellowish green color represents NBD-PS-coated SWCNT inside the cells. Original magnification - 63×2. Data are mean±s.d.

Furthermore, primary human monocyte-derived macrophages (HMDM) and human monocyte-derived dendritic cells (MDDC) also preferentially recognized and engulfed PS-coated SWCNT compared to PC-coated SWCNT (Fig. 6). The latter data thus confirm that the selective uptake of PS-functionalized SWCNT is a general phenomenon that is seen with phagocytes derived from different species. For the studies of human monocyte-derived phagocytes, flow cytometry was used to quantify the degree of *in vitro* uptake of NBD-PC- versus NBD-PS-coated nanotubes (Fig. 6A, Fig. 6B), and these data were confirmed using confocal microscopy (Fig. 6C) and TEM evaluation of non-NBD labeled phospholipid-coated SWCNT (data not shown).

### Interaction of PS-coated SWCNT with HeLa cervical carcinoma cells and SH-SY5Y neuroblastoma cells

**Figure 7:**
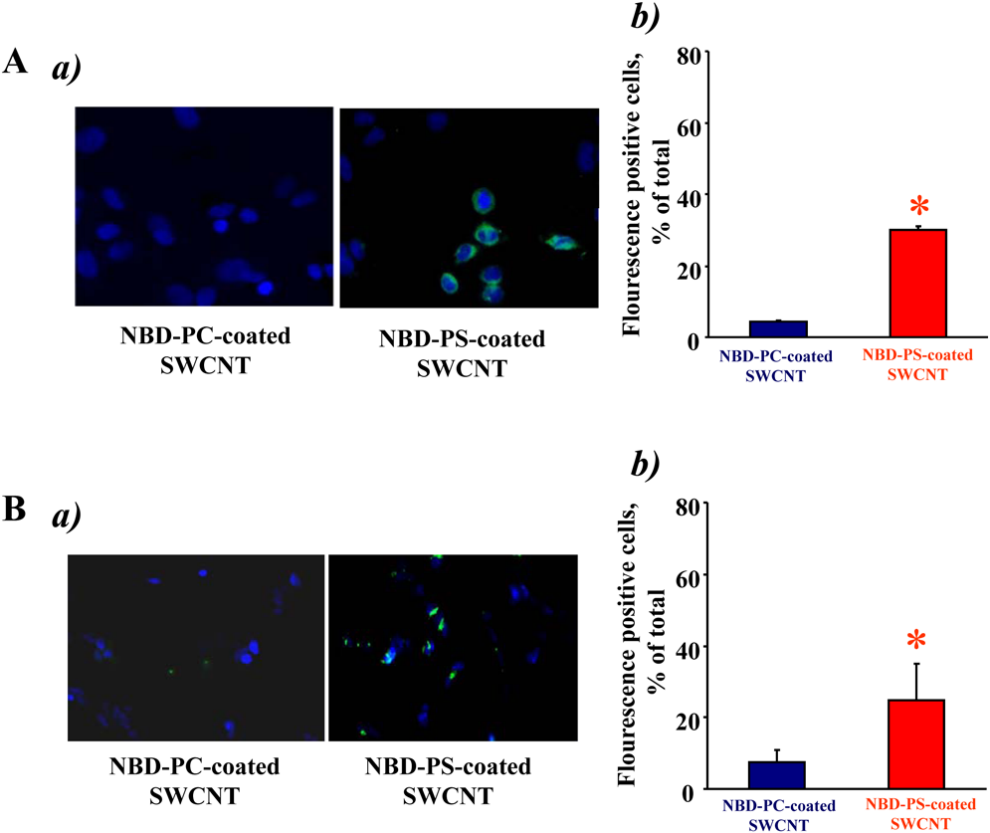
HeLa cervical carcinoma cells and SH-SY5Y neuroblastoma cells interact with PS-coated (but not with PC-coated) SWCNT. A. *In vitro* assessment of uptake of SWCNT coated with NBD-PS or NBD-PC by HeLa cells. a) Overlay of blue and green fluorescence images. b) Quantitative evaluation of cell with engulfed SWCNT. Data are mean±s.d., n = 4. *p<0.05, NBD-PS-coated SWCNT vs NBD-PC-coated SWCNT. B. *In vitro* assessment of uptake of SWCNT coated with NBD-PS or NBD-PC by SH-SY5Y neuroblastoma cells. a) Overlay of blue and green fluorescence images. b) Quantitative evaluation of cells with engulfed SWCNT. Data are mean±s.d., n = 4. *p<0.05, NBD-PS-coated SWCNT vs NBD-PC-coated SWCNT.

While ingestion of apoptotic cells is not absolutely specific to phagocytes, the rates of uptake by professional phagocytes - macrophages and microglial cells – are significantly higher compared to non-professional phagocytes (such as endothelial, epithelial cells and fibroblasts) [52]. Typical fluorescence micrographs of cervical carcinoma HeLa cells (Fig. 7Aa) and neuroblastoma SH-SY5Y cells (Fig. 7Ba) demonstrate preferential uptake of NBD-PS-coated SWCNT vs NBD-PC-coated SWCNT by both cell types. Quantitative assessments revealed that PS-coated SWCNT were ingested more actively than NBD-PC-coated SWCNT by HeLa cells, and SH-SY5Y cells, respectively (Fig 7Ab and Fig. 7Bb). However, uptake of NBD-PS-coated SWCNT by these cells was 2.4 times and 2.6 times less effective, respectively, than their ingestion by RAW 264.7 macrophages.

### Recognition of PS-coated SWCNT by alveolar macrophages *in vivo*

**Figure 8:**
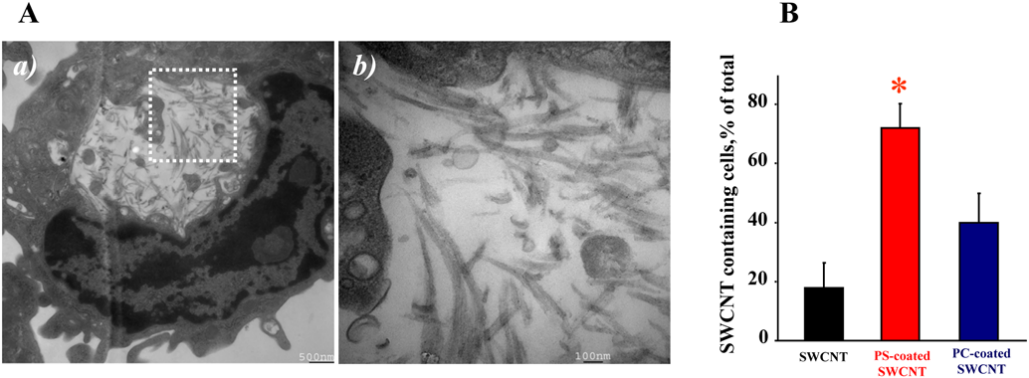
SWCNT functionalized with PS but not with PC engulfed *in vivo* by murine alveolar macrophages. A. a)Transmission electron micrographs of bronchoalveolar macrophages isolated from C57BL/6 mice exposed to PS-coated SWCNT; b) high magnification image of the area shown in (a). B. Alveolar macrophages preferentially phagocytoze PS-coated SWCNT in vivo. TEM images were used to calculate the percent of phagocytosis-positive macrophages. At least 150 cells were counted for each treatment. Data are mean±s.d., *p<0.05, PS-coated SWCNT *vs* non-coated or PC-coated SWCNT. C57BL/6 female mice, 7–8 weeks old, were exposed to SCWNT (40 µg/mouse) by pharyngeal aspiration. Twenty-four hours following exposure, mice were sacrificed using sodium pentobarbital. Mice were lavaged with sterile PBS. The lavage fractions were pooled and centrifuged to obtain the cellular fraction. Cells were then fixed for TEM.

Because PS is an important recognition signal for alveolar macrophages in the lung [15], we reasoned that PS-coated SWCNT could be effectively phagocytozed *in vivo*. To this end, we used an established mouse model of SWCNT pulmonary exposure through pharyngeal aspiration [36]. Mice were exposed to PS-coated-, PC-coated-, or non-coated SWCNT using procedures described previously [53]. A high magnification TEM image of alveolar macrophages obtained from animals exposed to PS-coated SWCNT clearly demonstrates the presence of “bamboo shoot”-like material (Fig. 8A). We also performed quantitative assessments of phagocytosis by counting SWCNT-positive alveolar macrophages using TEM images. Macrophages with at least one nanoparticle engulfed were considered as phagocytosis-positive. The data are presented as a plot of percentage of active phagocytes for each condition (Fig. 8B). BAL obtained from PS-coated SWCNT–exposed mice revealed 72±8.4% of phagocytosis-positive alveolar macrophages. In contrast, only 40±10% and 18±8% of macrophages isolated from mice exposed to PC/SWCNT or non-coated SWCNT contained nanoparticles. Thus, PS-coated SWCNT were ingested at a significantly higher rate in vivo as compared to PC-coated or non-coated SWCNT by alveolar macrophages.

## Discussion

Specific interfacing of SWCNT with phagocytic cells of the immune system – macrophages, microglia, and dendritic cells - is important for several reasons. The first one is that SWCNT can be used for simultaneous targeted delivery of several different regulators/inhibitors with a potential to release them in temporally and spatially predetermined ways to control the bioactivity of a specific cell population during physiologically critical events. As macrophages can host a number of pathogens [54], [55], nanocarriers can also be used for specific delivery of pro-apoptotic agents to aid in the defense against intracellular pathogens [56]. Furthermore, macrophages and microglia are the major executors of pro-/anti-inflammatory responses, and - along with antigen-presenting dendritic cells - are important components of immune reactions [57]–[60]. Specific targeting of cargoes/regulators to these cells could be exploited for therapeutic regulation of numerous immune functions, including the enhancement of immune responses to prophylactic or therapeutic allergen-specific vaccines through the coupling of allergens to nanocarriers. Finally, SWCNT are among the most commonly used nanomaterials with explosively expanding research and commercial applications [61]. Because the production and employment of industrial quantities of SWCNT are becoming a reality, health risk concerns, particularly due to occupational and environmental exposures, are emerging [62]. Not only an unusually large surface area, but also unique physical and chemical characteristics, redox features as well as significant decoration with transition metals alert to a possibility of unanticipated bioresponses resulting from interactions of SWCNT with cells, tissues, and biofluids. In fact, recent studies have demonstrated significant pulmonary and cardiovascular toxicity of SWCNT associated with a robust inflammatory response and early onset of fibrotic transition in mice [53], [63], [64]. In this context, the enhancement of phagocytic recognition and uptake of SWCNT through PS-functionalization may be important in order to reduce the potential cytotoxicity of SWCNT.

Understanding of major principles of particle recognition by macrophages has long been a controversial issue. Because non-functionalized nanoparticles are prone to aggregation, sonication of non-coated SWCNT was performed before adding them to cells. Under these conditions, no significant uptake of non-coated SWCNT by RAW 264.7 macrophages occurred during the 2 h incubation period. In contrast, Dumortier et al. [65] have recently reported that SWCNTs tend to re-aggregate and form large clusters that are eventually (after 24 h of co-incubation) phagocytozed by professional macrophages. It is likely that macrophage uptake of big clusters of SWCNT formed during prolonged incubation times is related to the reduced solubility of these non-coated nanotubes. It has been also reported that geometry and shape act as determinants of particle recognition [66]. Several studies have demonstrated that functionalized SWCNT are recognizable by cells and taken up through endocytosis-dependent pathways [67], [68]. By contrast, we and others reported that non-functionalized SWCNT are neither effectively recognized nor phagocytozed by macrophages [5], [53], [69]. The fact that PS-coating leads to recognition and uptake of SWCNT suggests that it is the lack of a recognition signal that is responsible for poor uptake of non-functionalized carbon nanotubes by phagocytes. A variety of specialized receptors on the macrophage surface have been implicated in recognition and tethering of different particles, including ultra-fine particles [64], [67], [70]–[72]. Recent studies identified several novel macrophage receptors for which PS is a specific high-affinity ligand [25], [26], facilitating uptake of target cells with externalized PS. PC is not specifically recognized by these receptors and its presence on the cell surface does not enhance recognition and uptake of cells by macrophages. Therefore, we chose to use NBD-PC-coated SWCNT as controls in our comparative experiments on assessments of SWCNT uptake by macrophages. Further, we utilized Annexin V – a protein known to selectively bind to PS (but not to PC) and mask PS recognition and uptake by macrophages. The Annexin V mediated suppression of uptake of NBD-PS-coated SWCNT (and lack of fluorescence response from macrophages incubated in the presence of NBD-PS coated SWCNT pre-treated with Annexin V) was employed as an additional specific control of PS-dependent recognition and uptake. Our data indicate that eclipsing of PS with its specific ligand, Annexin V, completely blocks SWCNT recognition by macrophage cell lines and primary phagocytes. Most notably, these PS-dependent recognition patterns are realized *in vivo* whereby alveolar macrophages display enhanced uptake of PS-coated SWCNT during an inflammatory pulmonary response induced by aspiration exposure. Together, our *in vitro* and *in vivo* studies demonstrate that PS-functionalization renders nanotubes appetizing to phagocytes. We believe that analysis of SWCNT uptake by phagocytes based on the employment of fluorescently-labeled phospholipids (NBD-PS and NBD-PC) provided more quantitatively reliable data. In this case, however, the limitations of quantitative assessments might be due to a possibility that fluorescently-labeled phospholipids could also modify (promote or decrease) the uptake of these fluorescent phospholipid coated SWCNT by macrophage. To minimize this interference, mole fraction of fluorescently-labeled phospholipids in the mixture with non-labeled phospholipids of the same type in all experiments did not exceed 10 mol%. Notably, the TEM-based evaluations of phagocytotic activity towards SWCNT were in good agreement with independent assessments performed by confocal imaging of NBD-labeled phospholipids-coated SWCNT.

PS-induced responses initiate signaling cascades in macrophages, switching off their pro-inflammatory activation pattern, and turning on the production of anti-inflammatory cytokines and chemokines [15], [73]. This suggests that functionalization in general, and PS-coating in particular, may change not only recognition but also pro-/anti-inflammatory behavior of macrophages interacting with nanoparticles. In line with this, our experiments demonstrated that PS coating of SWCNT could be utilized not only for directing SWCNT to phagocytic cells but also as a regulator or “switch” affecting the profile of the produced and released cytokines *in vitro* and *in vivo* [16], [19]. Our studies show that PS-coating of SWCNT confers on them a “zip-code” address directing their recognition, engulfment, and uptake by professional phagocytes. Our results show that non-covalent attachment of cyt c to PS-coated SWCNTs, and subsequent release of cyt c inside macrophages using an endosome-disrupting agent, effectively activated caspase-3 in these cells, indicative of activation of apoptosis. These results are thus at variance with recent studies indicating that cellular uptake of functionalized nanotubes is independent of the nature of the functional group [9]. It must be noted, however, that the latter studies did not include professional phagocytes (macrophages) but rather a panel of non-phagocytic cell lines such as Jurkat, A549, HeLa cells, and so forth. Moreover, the studies by Kostarelos et al. [9] also indicated that the uptake in non-phagocytic cells could occur through passive penetration of the nanotubes through the plasma membrane. In contrast, one of the primary goals of our study was to develop approaches for targeting macrophages and other professional phagocytes by cytotoxic agents using nanotubes coated with PS as a specific ligand recognized by specialized plasma membrane receptors [25], [26]. We chose to use cyt c as a “death” signal. Cyt c is one of the key co-factors for the activation of apoptosis in mammalian cells [62] and is known to be a difficult cargo for targeted delivery into cells [6], [74], thus justifying the employment of cyt c as an appropriate “proof-of-principle” reagent. Our data show for the first time that PS-functionalized nanotubes could be used for selective delivery of specified cargoes (cyt c) into professional phagocytic cells, resulting in regulation of activity/survival of these cells.

Importantly, manipulating macrophage apoptosis can be a valuable therapeutic strategy in several diseases associated with the presence of intracellular pathogens in macrophages such as *Mycobacterium tuberculosis* and *Listeria monocytogenes*. Molloy et al. [75] showed that macrophage apoptosis resulted in reduced viability of intracellular mycobacteria. *L. monocytogenes* was reported to induce apoptosis in vitro and in vivo in a variety of cell types with the exception of macrophages which represent the predominant compartment of bacterial multiplication and die as a result of necrosis. Shifting the equilibrium from necrosis to apoptosis in *L. monocytogenes* infected macrophages is believed to constitute a promising therapeutic strategy [76]. Two other relevant examples are macrophage activation syndrome and rheumatoid arthritis - diseases where uncontrolled macrophage proliferation or macrophage resistance to apoptosis, respectively, represents important features of disease pathogenesis [77], [78].

Dai et al [79] have previously described the functionalization of SWCNT with a folate moiety for targeting of nanotubes to folate receptor-rich tumor cells in vitro, which could be considered for future cancer therapy. Mioskowski et al. [80] reported the self-assembly of several *synthetic* single-chain lipids around SWCNT to form supramolecular structures designed for the immobilization of histidine-tagged proteins; they did not investigate whether such nanotubes were ingested by cells. In addition, in a recent and elegant study, Liu et al. [81] reported on the attachment of an RGD peptide to nanotubes coated with polyethylene glycol (PEG) and subsequent in vivo administration to mice bearing integrin-positive tumors. Efficient targeting of the nanotubes to the tumors was achieved through this approach. The goal of our work was to employ the naturally occurring “eat-me” signal, PS as a specific ligand that is recognized by professional phagocytes. We provide evidence that the uptake is specific, since nanotubes functionalized with PC are not recognized as readily as PS-coated nanotubes and because the PS-binding protein, Annexin V, can prevent uptake of PS-coated nanotubes. Because the chemical cutting is associated with the production of oxidative negatively-charged defects in SWCNT (carboxy-groups [32]) it is possible that coating with phospholipids could be slightly different in precut and non-precut SWCNT. It should be noted, however, that binding of negatively charged PS (at pH 7.4) is unlikely to occur at the sites of negatively charged defects due to electrostatic repulsions. Previous studies from our and other laboratories have established high-affinity binding of cyt c with PS [82]. After the coating, binding of positively-charged cyt c was likely associated with abundant negatively-charged PS. We also provide evidence that the mechanism of uptake is specific and occurs through endocytosis. Finally, we show that a relevant cargo (pro-apoptotic cyt c) can be delivered into macrophages, and upon disruption of endosomes, the cargo is released and can perform its biological function (activation of the caspase cascade).

Taken together, our findings highlight a novel strategy for the controlled delivery of relevant cargoes into specific cell populations. Further developments of the novel principle established in the current study, i.e. PS-coating of SWCNT for recognition and ingestion, may exploit cargoes covalently-conjugated with specific linkers that are hydrolysable extracellularly by activated macrophages (eg. superoxide-sensitive linkers) or intracellularly (eg. esterase-sensitive conjugates) for targeted delivery and release to regulate life-span and activity of phagocytes.

## Supporting Information

Figure S1 TEM images of bare SWCNT (A) and PS-coated SWCNT (B) deposited on mica substrates.
(5.93 MB TIF)

Figure S2 Viability of RAW264.7 macrophages exposed to SWCNT. RAW 264.7 macrophages were seeded at 1×104 cells/ well on 96 well-plate 12 hrs before treatment. Cells were incubated with non-coated SWCNT or SWCNT coated with either PS or PC in serum and phenol red free DMEM containing 100 µM DTPA for up to 4 hrs. At the end of incubation, the culture medium was collected, centrifuged to remove SWCNT and used for LDH assay with CytotoxOne kit. Data are mean±s.d., n = 3.
(0.50 MB TIF)

Figure S3 PS-coated SWCNT effectively deliver cyt c into RAW264.7 macrophages, and activate apoptotic pathways (caspase 3/7). RAW264.7 macrophages were seeded at 0.5×105 cells/well 12 hrs prior to treatment. Cells were incubated with differently functionalized SWCNT in serum and phenol red free DMEM containing 100 µM DTPA for 1 hr at 37°C. At the end of incubation, cell were washed to remove SWCNT and treated with chloroquine (100 µM) in complete culture medium at 37 °C for 15 min. After that, cells were washed with complete DMEM and additionally incubated for 8 hrs. Cells were stained in binding buffer containing annexin V (0.5 µg/ml) for 5 min and counter-stained with Hoechst 33342 for nuclei at room temperature. The cells were examined under a Nikon ECLIPSE TE 200 fluorescence microscope (Tokyo, Japan) equipped with a digital Hamamatsu CCD camera (C4742-95-12NBR) and analyzed using the MetaImaging Series™ software version 4.6 (Universal Imaging Corp., Downingtown, PA). A minimum of 300 macrophages were counted per experimental condition. Data are mean±s.d. (* p<0.01 vs untreated). Insert: Typical fluorescence image of RAW264.7 macrophages treated with cyt c/PS-coated SWCNT (staining with annexin V).
(1.39 MB TIF)

Table S1 (0.03 MB DOC)
