# Correction: Phosphatidylserine Targets Single-Walled Carbon Nanotubes to Professional Phagocytes *In Vitro* and *In Vivo*

**DOI:** 10.1371/annotation/1801d3b3-2082-4eb7-913b-b93e1fe4c219

**Published:** 2009-05-11

**Authors:** Nagarjun V. Konduru, Yulia Y. Tyurina, Weihong Feng, Liana V. Basova, Natalia A. Belikova, Hülya Bayir, Katherine Clark, Marc Rubin, Donna Stolz, Helen Vallhov, Annika Scheynius, Erika Witasp, Bengt Fadeel, Padmakar D. Kichambare, Alexander Star, Elena R. Kisin, Ashley R. Murray, Anna A. Shvedova, Valerian E. Kagan

The following funding information was omitted: This work was supported by NANOMMUNE No 214281 FP7-NMP-2007-SMALL-1.

