# Supplementary figures and images for: Phosphatidylserine Targets Single-Walled Carbon Nanotubes to Professional Phagocytes In Vitro and In Vivo
Source: PLoS One. 2009 Feb 9;4(2):e4398. doi: 10.1371/journal.pone.0004398 (PMC2634966; doi:10.1371/journal.pone.0004398)

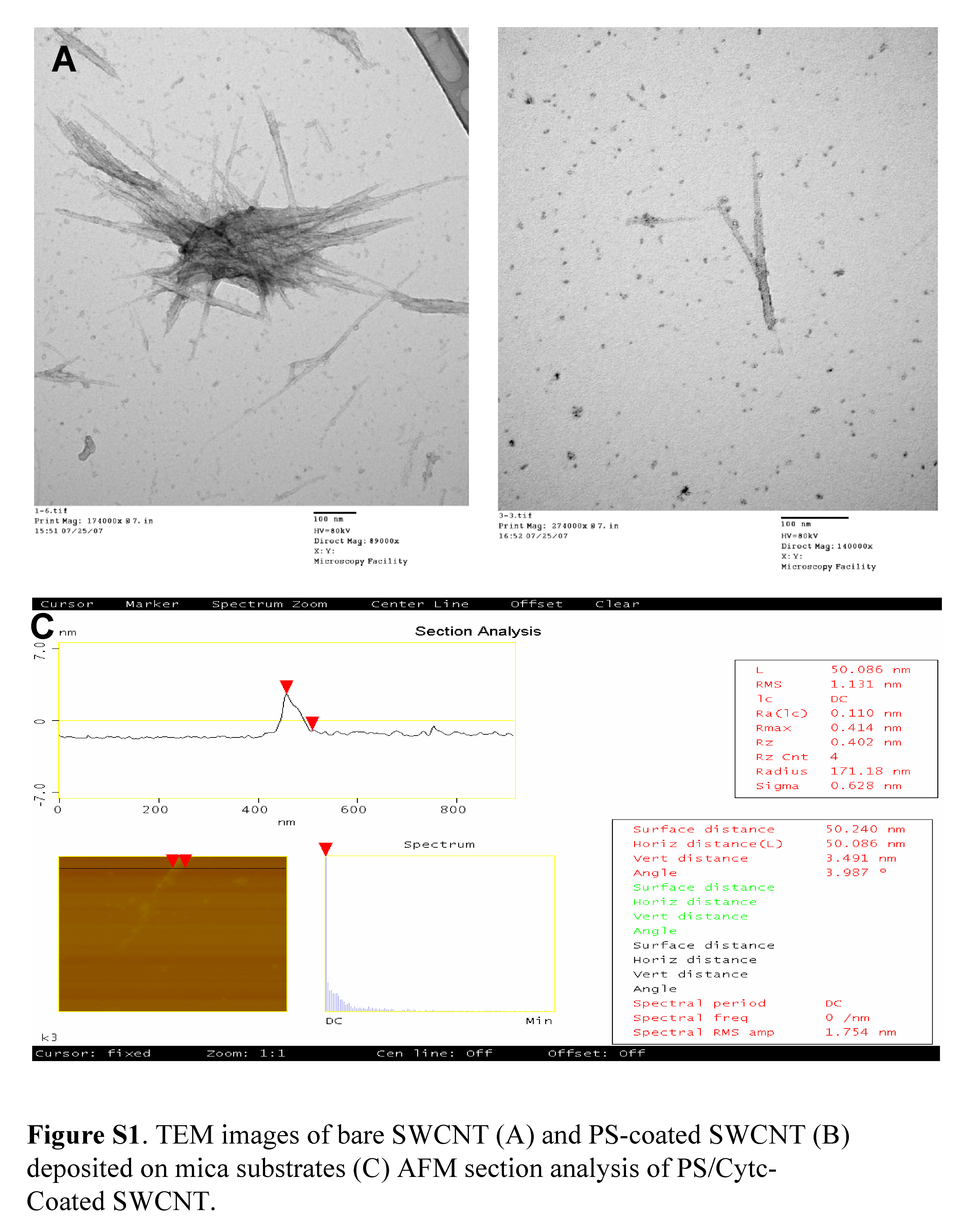

Supplement: Figure S1 — TEM images of bare SWCNT (A) and PS-coated SWCNT (B) deposited on mica substrates. (5.93 MB TIF) [file pone.0004398.s001.tif]

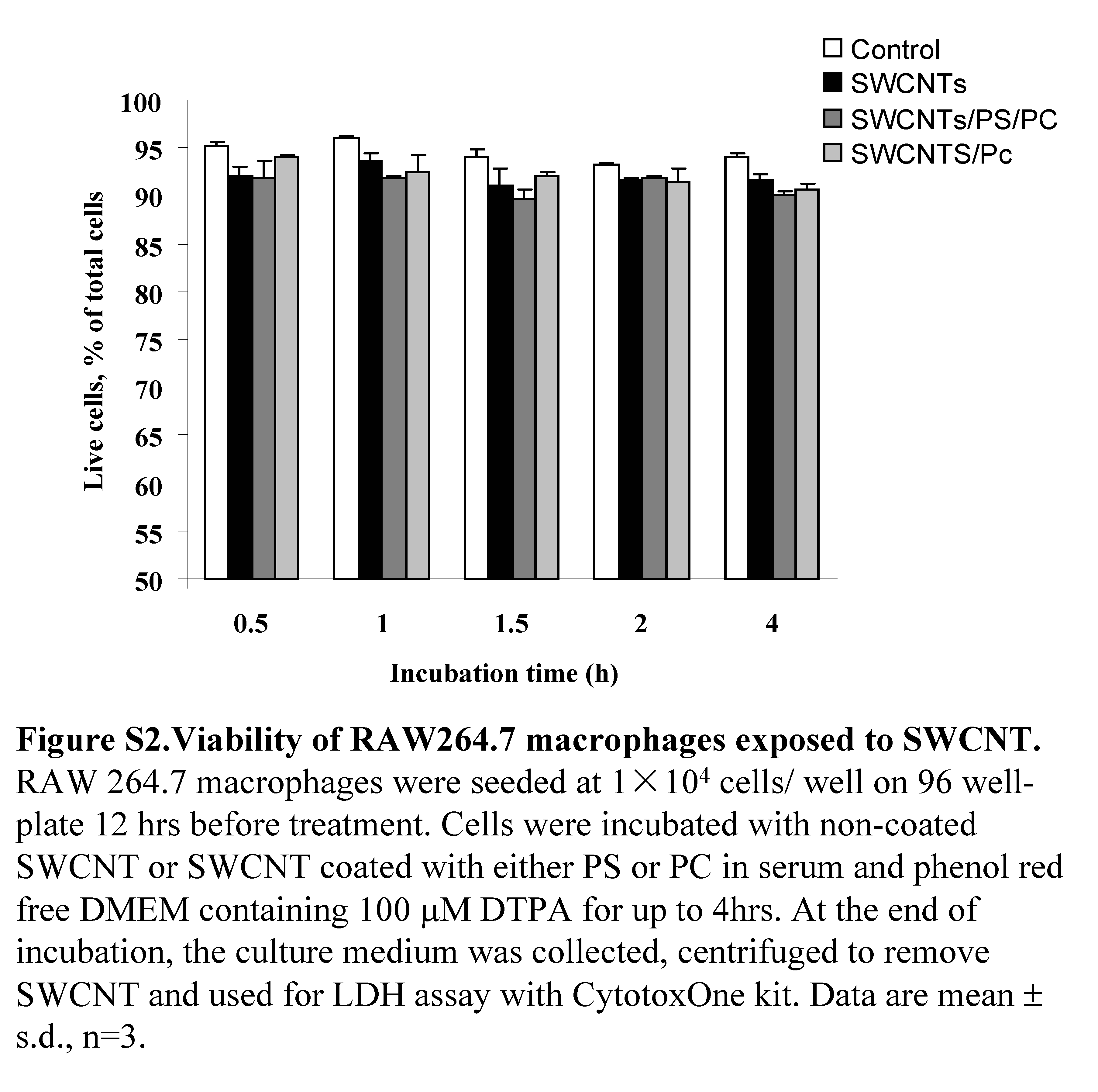

Supplement: Figure S2 — Viability of RAW264.7 macrophages exposed to SWCNT. RAW 264.7 macrophages were seeded at 1×10^4^ cells/ well on 96 well-plate 12 hrs before treatment. Cells were incubated with non-coated SWCNT or SWCNT coated with either PS or PC in serum and phenol red free DMEM containing 100 µM DTPA for up to 4 hrs. At the end of incubation, the culture medium was collected, centrifuged to remove SWCNT and used for LDH assay with CytotoxOne kit. Data are mean±s.d., n = 3. (0.50 MB TIF) [file pone.0004398.s002.tif]

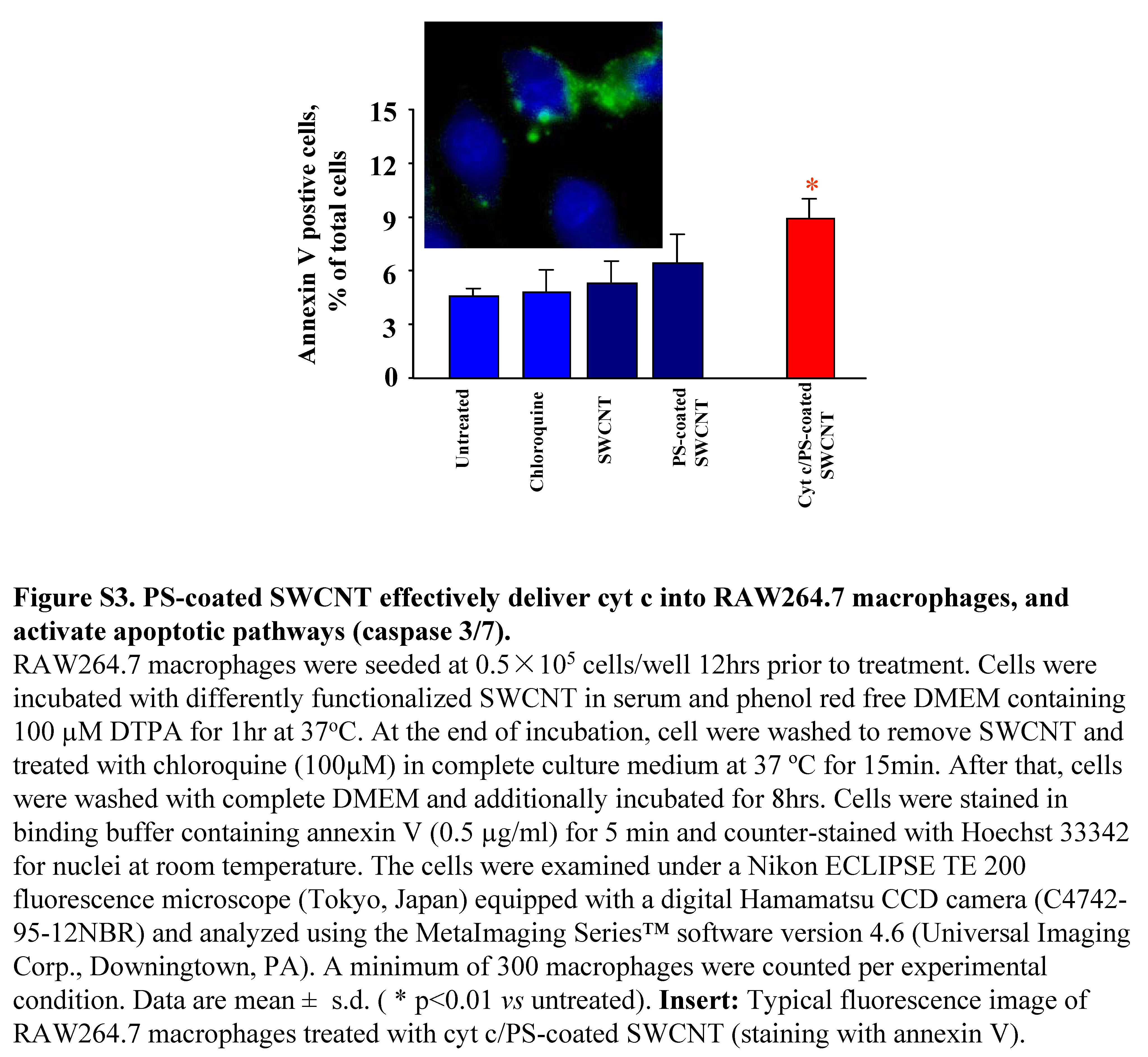

Supplement: Figure S3 — PS-coated SWCNT effectively deliver cyt c into RAW264.7 macrophages, and activate apoptotic pathways (caspase 3/7). RAW264.7 macrophages were seeded at 0.5×10^5^ cells/well 12 hrs prior to treatment. Cells were incubated with differently functionalized SWCNT in serum and phenol red free DMEM containing 100 µM DTPA for 1 hr at 37°C. At the end of incubation, cell were washed to remove SWCNT and treated with chloroquine (100 µM) in complete culture medium at 37 °C for 15 min. After that, cells were washed with complete DMEM and additionally incubated for 8 hrs. Cells were stained in binding buffer containing annexin V (0.5 µg/ml) for 5 min and counter-stained with Hoechst 33342 for nuclei at room temperature. The cells were examined under a Nikon ECLIPSE TE 200 fluorescence microscope (Tokyo, Japan) equipped with a digital Hamamatsu CCD camera (C4742-95-12NBR) and analyzed using the MetaImaging Series™ software version 4.6 (Universal Imaging Corp., Downingtown, PA). A minimum of 300 macrophages were counted per experimental condition. Data are mean±s.d. (* p<0.01 vs untreated). Insert: Typical fluorescence image of RAW264.7 macrophages treated with cyt c/PS-coated SWCNT (staining with annexin V). (1.39 MB TIF) [file pone.0004398.s003.tif]
