# Supplementary material for: Phosphatidylserine Targets Single-Walled Carbon Nanotubes to Professional Phagocytes In Vitro and In Vivo
Source: PLoS One. 2009 Feb 9;4(2):e4398. doi: 10.1371/journal.pone.0004398 (PMC2634966; doi:10.1371/journal.pone.0004398)
Supplement: Table S1 — (0.03 MB DOC) [file pone.0004398.s004.doc]

Supplementary Table 1. Physico-chemical characteristics of SWCNTs.

Sample ID Zeta pH Size Elemental Carbon

Potential (mV) distribution (nm) content (ng/µl)

SWCNT -42.3 ± 0.9 6.23 135 ± 3 36 ± 6

SWCNT/PS -50.4 ± 0.9 7.14 162 ± 5 59 ± 11

SWCNT/PC -48.2 ± 0.2 7.02 155 ± 1 75 ± 16

SWCNT/PS-NBD -46.4 ± 0.5 6.93 153 ± 2

SWCNT/PC-NBD -34.9 ± 4.5 6.94 169 ± 5

SWCNT/cyt c -38.8 ± 1.8 7.03 156 ± 3

SWCNT/cyt c/PS -49.5 ± 3.5 7.02 164 ± 2 96 ± 19

SWCNT/cyt c/PC -52.2 ± 3.3 7.14 171 ± 3

All Data are mean ± s.d. (of measurements).
